# Cyclometalated gold(iii) complexes: noticeable differences between (N,C) and (P,C) ligands in migratory insertion†
†Electronic supplementary information (ESI) available: Detailed experimental conditions and procedures, analytical data, theoretical details (PDF), crystallographic data for compounds **5A-H_2_O** and **5A-Cl**, and DFT optimized structures (XYZ). CCDC 1574199 and 1574200. For ESI and crystallographic data in CIF or other electronic format see DOI: 10.1039/c7sc04899h


**DOI:** 10.1039/c7sc04899h

**Published:** 2018-03-26

**Authors:** Jordi Serra, Pau Font, E. Daiann Sosa Carrizo, Sonia Mallet-Ladeira, Stéphane Massou, Teodor Parella, Karinne Miqueu, Abderrahmane Amgoune, Xavi Ribas, Didier Bourissou

**Affiliations:** a QBIS-CAT Group , Institut de Química Computacional i Catàlisi (IQCC) , Departament de Química , Universitat de Girona , Campus Montilivi , Girona , E-17003 , Catalonia , Spain . Email: xavi.ribas@udg.edu; b CNRS/UNIV PAU & PAYS ADOUR , Institut des Sciences Analytiques et de Physico-Chimie pour l'Environnement et les Matériaux (IPREM, UMR 5254) , Hélioparc , 2 Avenue du Président Angot , 64053 Pau Cedex 09 , France; c Institut de Chimie de Toulouse (FR 2599) , 118 Route de Narbonne , 31062 Toulouse Cedex 09 , France; d Servei de RMN , Facultat de Ciències , Universitat Autonòma de Barcelona , Campus UAB , Bellaterra E-08193 , Catalonia , Spain; e CNRS , Université Paul Sabatier , Laboratoire Hétérochimie Fondamentale Appliquée (LHFA, UMR 5069) , 118 Route de Narbonne , 31062 Toulouse Cedex 09 , France . Email: dbouriss@chimie.ups-tlse.fr

## Abstract

Gold(iii) complexes are garnering increasing interest for opto-electronic, therapeutic and catalytic applications. But so far, very little is known about the factors controlling their reactivity and the very influence of the ancillary ligand. This article reports the first comprehensive study on this topic. The reactivity of a cationic (N,C) gold(iii) complex, namely **1A**, towards ethylene has been thoroughly studied and compared with that of the related (P,C) complex **1C**. A cationic gold(iii) complex **5A** resulting from double insertion of ethylene was selectively obtained. Complex **5A** was found to be remarkably stable. It was trapped with chloride and fully characterized. In marked contrast to that observed with **1C**, no β-H elimination or linear-to-branched rearrangement of the alkyl chain occurred with **1A**. The energy profile for the reactions of **1A** with ethylene has been comprehensively investigated computationally, and the influence of the ancillary ligand has been precisely delineated. Because nitrogen is a weaker donor than carbon (and phosphorus), the (N,C) ligand is very electronically dissymmetric, much more than the (P,C) ligand. This makes the two reactive sites at gold quite different, which noticeably influences the competition between migratory insertion and β-H elimination, and actually changes the outcome of the olefin insertion at gold. This study provides valuable insight into the influence of ancillary ligands on gold(iii) reactivity, something critical to further develop Au(iii) and Au(i)/Au(iii) catalysis.

## Abstract 2

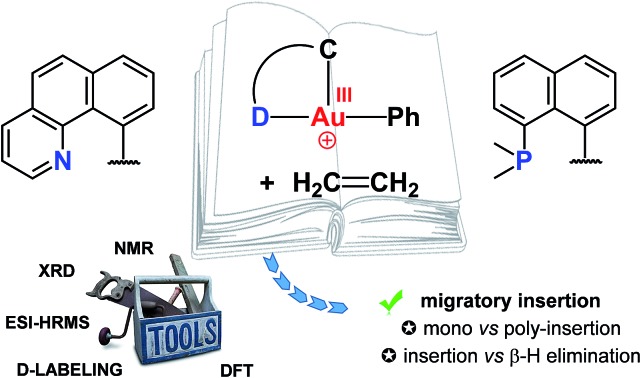
Gold(iii) complexes are garnering increasing interest for opto-electronic, therapeutic and catalytic applications.

## Introduction

The past few years have witnessed spectacular development in gold(iii) chemistry.^1^–^4^ Au(iii) complexes display very interesting luminescence properties^5^,^6^ and biological activities,^7^,^8^ and increasing efforts are made to develop their opto-electronic and therapeutic applications. Au(iii) complexes also show unique catalytic properties, for the electrophilic activation of π–CC bonds and carbonyl compounds, and as intermediates in Au(i)/Au(iii) redox cycles.^3^,^9^–^11^

**Scheme 1:**
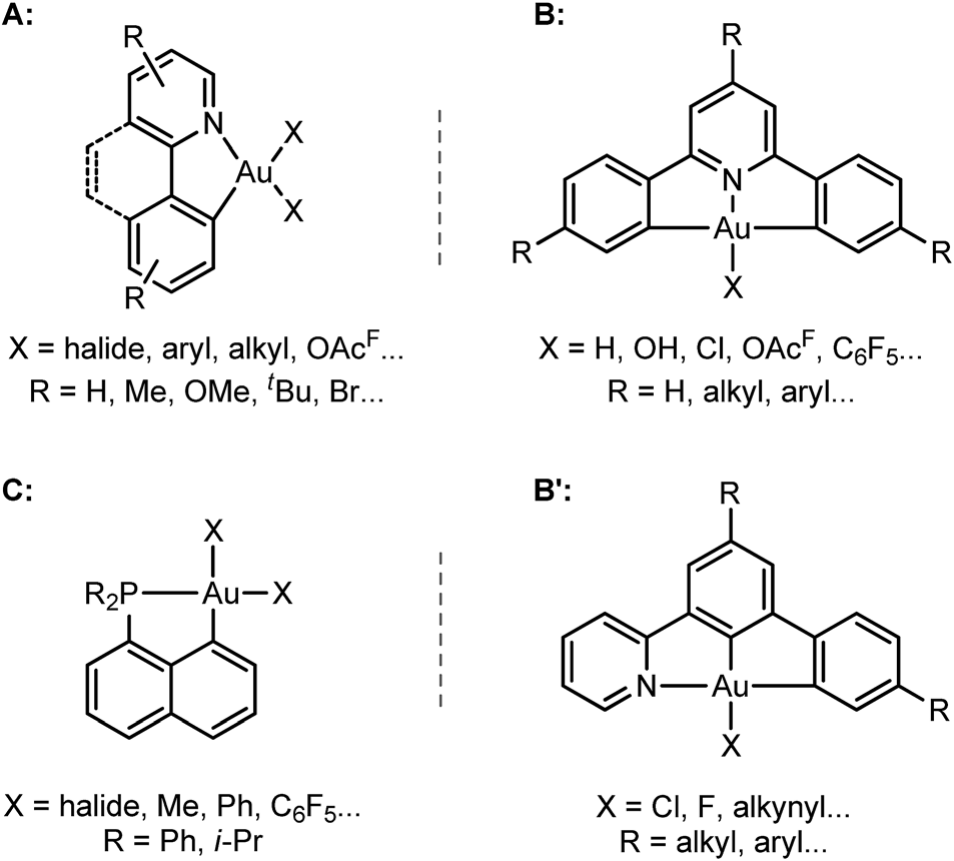
General structures of (N,C), (C,N,C), (N,C,C) and (P,C) gold(iii) complexes **A–C**.

Hard C- and N-based ligands occupy a forefront position in gold(iii) chemistry.^1^,^12^ In particular, (N,C) cyclometalated complexes **A**, and (C,N,C) and (N,C,C) pincer complexes **B** and **B′** offer a unique balance between stability and properties (Scheme 1).^6^,10c,d,^13^–^15^ Strikingly, soft P donors have also proved recently to efficiently stabilize gold(iii) species.^16^ (P,C) cyclometalated gold(iii) complexes **C** (which are readily available by chelate-assisted oxidative addition of C–X bonds)16*a* have been shown to display rich reactivity.16b–f Unprecedented elementary organometallic reactions such as migratory insertion of alkenes16b,c,e and β-hydride elimination16*d* have been evidenced, enlarging the portfolio of chemical transformations of gold complexes.^2^

To progress and develop further the chemistry of gold(iii) complexes, it is critical to better understand how their properties and reactivity are influenced by ancillary ligands. It was precisely the focus of this work to bridge the gap between widespread (N,C) gold(iii) complexes and recently introduced (P,C) gold(iii) species, and to try to answer the following questions:

(i) Is the unprecedented reactivity recently evidenced with (P,C) gold(iii) complexes specific to this ligand set or is it general? In other words, are (N,C) gold(iii) complexes also prone to migratory insertion and β-H elimination?

(ii) What is the precise influence of the ancillary ligand on reactions with alkenes? Does it affect the kinetics and thermodynamics? Does it change the mechanism and/or fate of the reaction?

**Scheme 2:**
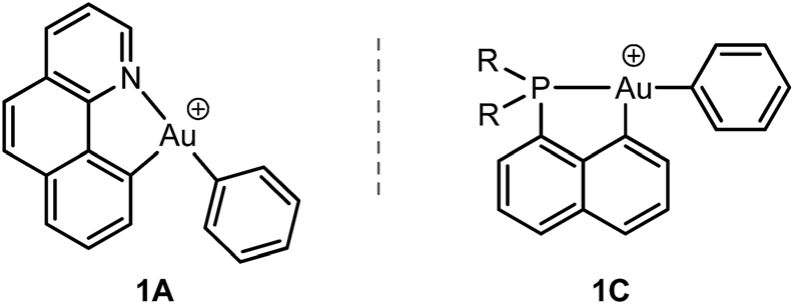
Structures of the (N,C) and (P,C) cationic gold(iii) complexes studied and compared in this work.

To this end, we studied a cationic tricoordinate (N,C) gold(iii) complex, namely [(N,C)AuPh]^+^**1A** (Scheme 2), and we report here a detailed investigation of its reactivity towards ethylene. This study draws some analogies with the related [(P,C)AuPh]^+^ complex **1C**,16*e* but also reveals noticeable differences. The weaker donicity of N *versus* P makes the (N,C) ligand much more electronically dissymmetric than the (P,C) ligand. As shown by detailed DFT investigations of the reaction profiles, these electronic properties noticeably influence the balance between the different reaction paths and eventually change the outcome of the ethylene insertion at gold. To the best of our knowledge, this is the first time that the impact of ancillary ligands on gold(iii) reactivity is thoroughly explored.

## Results and discussion

### Reaction of the [(N,C)AuPh]^+^ complex **1A** with ethylene

**Scheme 3:**
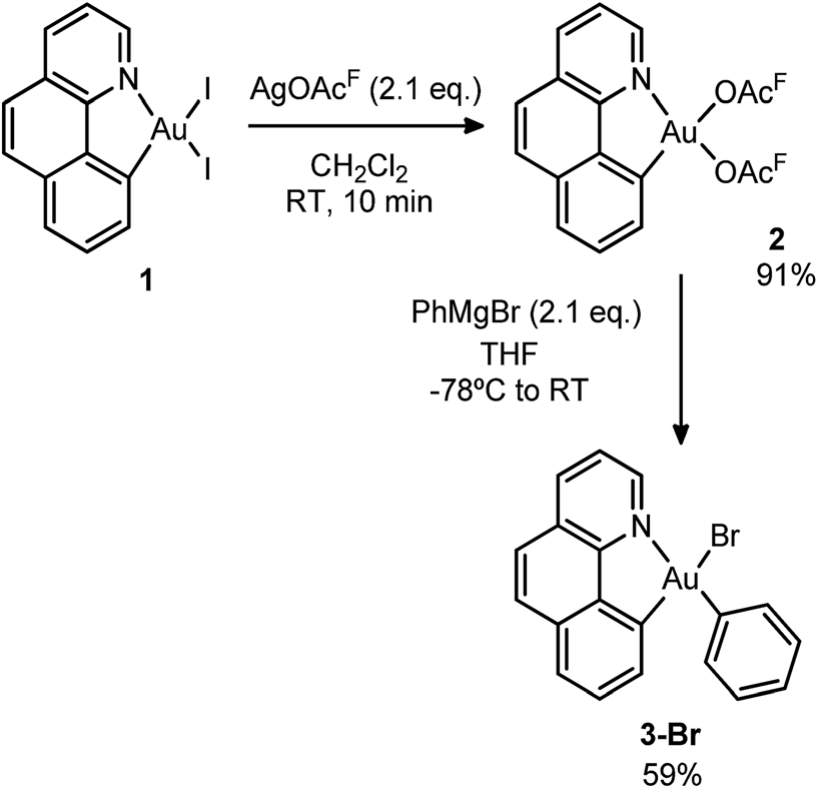
Synthesis of the Au(iii) complex **3-Br**, precursor to **1A**.

Initially, we attempted the selective mono-arylation of the [(N,C)AuI_2_] complex **1**, which was recently prepared *via* C_AR_–I oxidative addition to Au(i),13*f* following the same synthetic procedure as that described for the [(P,C)AuI_2_] complex.16*e* However, treatment of 1 with PhMgBr led to an intractable mixture of products. Hopefully, exchange of iodines at gold for more labile trifluoroacetate groups (complex **2**) allowed for the synthesis of the desired [(N,C)AuBrPh] complex **3-Br**(Scheme 3), applying the conditions reported by Tilset and co-workers.^17^ Complex **3-Br** was isolated as a white powder in 59% yield after column chromatography and characterized by NMR spectroscopy and high-resolution mass spectrometry (HRMS).^18^

**Scheme 4:**
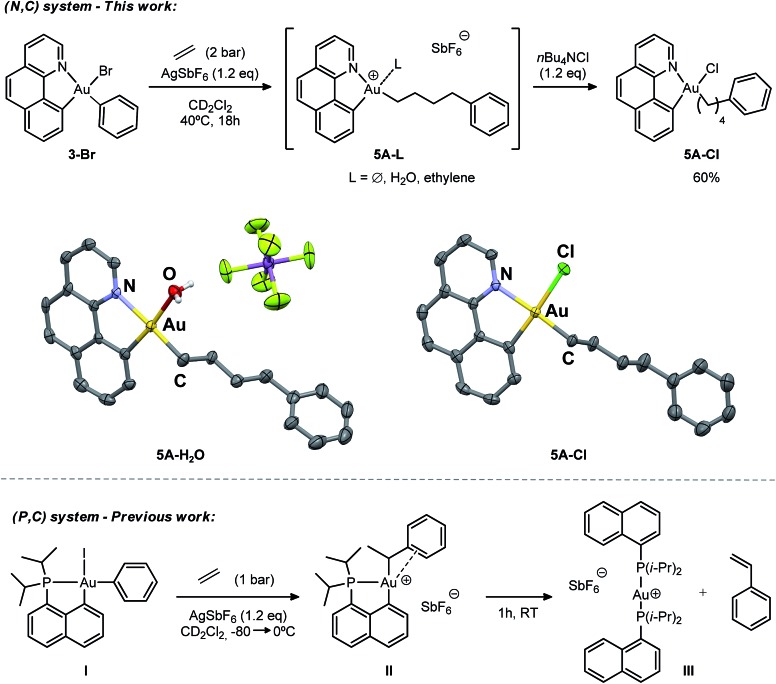
Reactions of the (N,C) and (P,C)-ligated gold(iii) complexes **1A** and **1C** with ethylene, and the molecular view of **5A-H_2_O** and **5A-Cl** with thermal ellipsoids drawn at the 50% probability level (hydrogen atoms have been omitted for clarity).

The cationic [(N,C)AuPh]^+^ complex **1A** was then generated by abstracting the bromide at the Au(iii) atom with AgSbF_6_ in dichloromethane under ethylene pressure (2 bar). No changes were observed within 72 h at room temperature, whereas upon heating at 40 °C for 18 h, complete conversion into a new species with aliphatic signals above *δ* 1.8 ppm was achieved (Scheme 4). Moreover, crystals formed spontaneously in small amounts inside the NMR tube, and X-ray diffraction analysis revealed double insertion of ethylene plus coordination of one molecule of adventitious water (Scheme 4). At this point, aiming at generating a more stable neutral four-coordinate gold(iii) complex to allow for easier handling and characterization,16d,e we quenched the reaction crude with *n*Bu_4_NCl. A white solid was isolated in 60% yield and characterized by multinuclear NMR spectroscopy and HRMS as **5A-Cl**. The relative integration of the aromatic and aliphatic signals in the ^1^H NMR spectrum (13 *versus* 8H), and the peak at *m*/*z* 508.1346 corresponding to the mass of [(N,C)AuPh]^+^ augmented by two ethylene molecules, corroborated the double insertion into the Au–Ph bond. The solid-state structure of the complex **5A-Cl** was also determined by X-ray crystallography.^18^ The gold atom sits in a square-planar environment formed by the (N,C) chelate ligand, the Cl atom and the (CH_2_)_4_Ph chain located *cis* to the aryl moiety.

The stability of the cationic gold complex **5A** generated under these conditions contrasts with the instability of **1A**. In the absence of ethylene, the latter fully decomposes within 6 h in solution at room temperature into several side-products and metallic gold. At this stage, and without knowing the precise structure of **5A** due to its ill-defined ^1^H NMR spectrum (the fourth coordination site at gold may be occupied by the SbF_6_ counter anion, the pendant Ph ring, an H_2_O or ethylene molecule), it is difficult to rationalize this difference.

The double addition of ethylene to complex **1A** is reminiscent of the insertion of two norbornene (NB) molecules into the Au–Me bond of the (P,C)-ligated gold(iii) dimethyl complex.16b,c In this process, the insertion of a second NB into the Au–C_norbornyl_ bond gives access to a more thermodynamically stable complex, with the Au–C_norbornyl_ bond at the *trans* position relative to phosphorus. In a likewise manner, the strong *trans* influence of the benzoquinolinic carbon in **1A** triggers the migration of the alkyl group by insertion of a second ethylene molecule at the *cis* position relative to the aryl moiety (see DFT calculations below). In spite of the presence of a hydrogen atom at a β position and the tendency of cationic low-coordinate gold(iii) alkyl complexes to undergo β-H elimination,16*d* complex **5A** shows unusual thermal stability and no formation of styrene or higher olefins was detected during the course of the reaction. This is in stark contrast to the behaviour of the [(P,C)AuPh]^+^ complex **1C**, where, after ethylene insertion, a β-H elimination and re-insertion sequence takes place leading to the gold(iii)–arene complex **II**, which after 1 h at room temperature eventually evolves into the linear gold(i) complex **III** by reductive elimination, styrene release and ligand redistribution (Scheme 4, bottom).16*e*

### D-labeling experiment

**Scheme 5:**
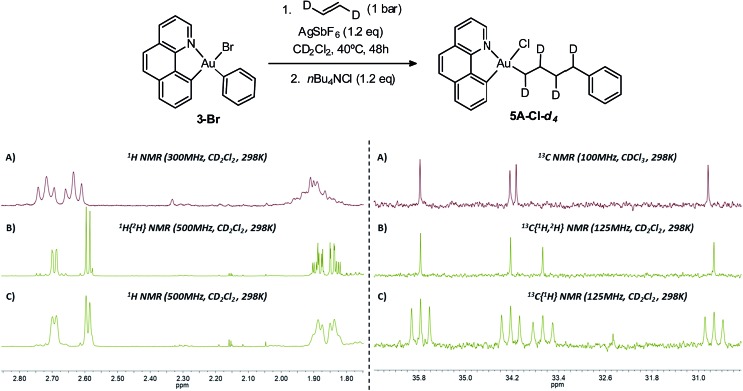
D-labeling experiment using *trans*-ethylene-*d*_2_ affording selectively the **5A-Cl-*d*_4_** product. Aliphatic ^1^H and ^13^C NMR signals of **5A-Cl** (A) and **5A-Cl-*d*_4_** (B, C), as obtained by the analysis of the reaction crudes. In the case of the complex derived from *trans*-ethylene-*d*_2_, deuterium-decoupled (B) and deuterium-coupled (C) NMR analyses are shown.

To assess the possible occurrence of β-H elimination in competition with ethylene insertion, a D-labeling experiment was carried out using *trans*-ethylene-*d*_2_ (Scheme 5). The reaction conditions have been adapted to enable the use of *trans*-ethylene-*d*_2_ which was supplied by Cluzeau Info Labo in a lecture bottle (1 atm, 95.2% D). Using an atmospheric pressure of ethylene, complete conversion was achieved after two ethylene refillings and 2 × 24 hours heating at 40 °C. The reaction was then repeated with *trans*-ethylene-*d*_2_ using the same experimental conditions.^18^ The reaction mixture was then trapped with *n*Bu_4_NCl and analysed by NMR spectroscopy. The ^1^H NMR spectrum shows the expected integration and pattern for the Au(CHD)_4_Ph enchainment (Scheme 5): the two terminal CHD moieties resonate as doublets compared to triplets for the non-deuterated complex (**5A-Cl**). Consistently, the corresponding ^13^C NMR signals all appear as 1 : 1 : 1 triplets in the ^13^C{^1^H} NMR spectrum. There is very little sign of H/D scrambling, if any, confirming that β-H elimination does not occur to a significant extent under these conditions and that ethylene insertion prevails.

### Synthesis and characterization of the [(N,C)Au(*n*Bu)]^+^ complex **6A**

**Scheme 6:**
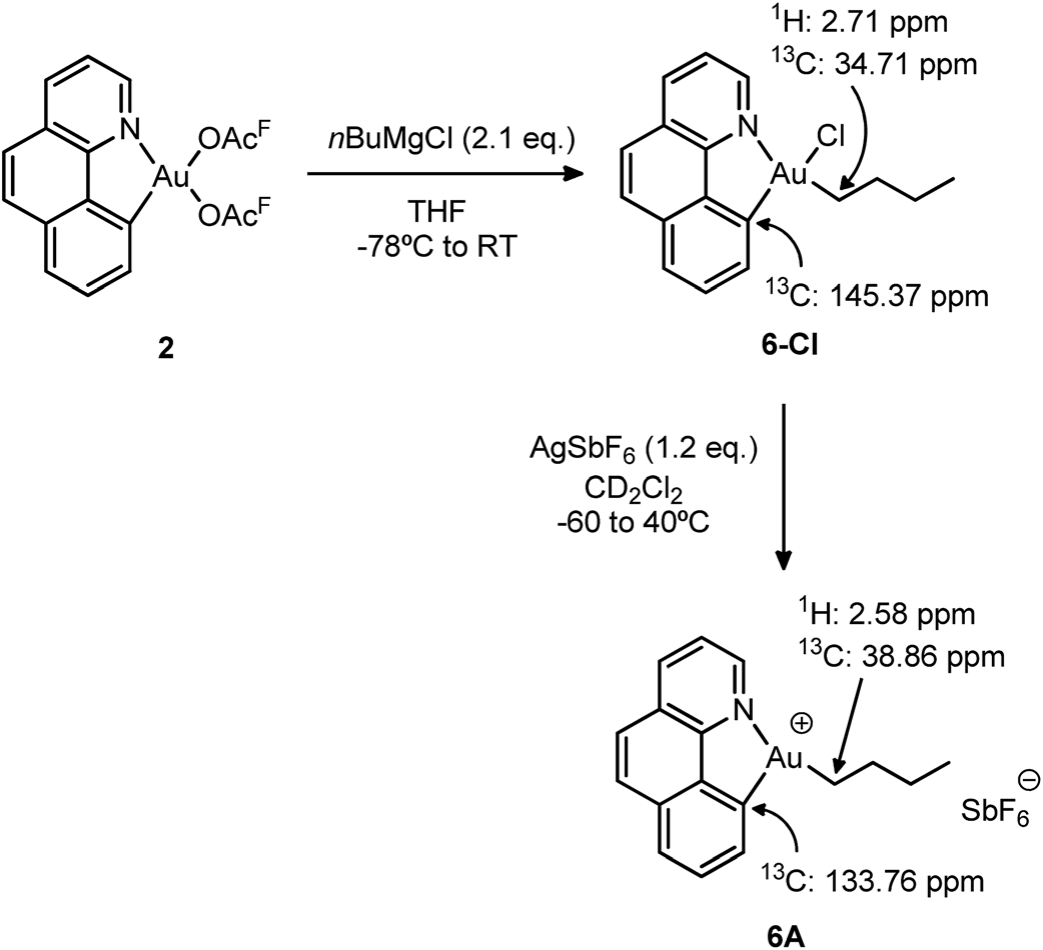
Synthesis of the *n*-butyl complexes **6-Cl** and **6A** and the most diagnostic ^1^H and ^13^C NMR shifts.

To gain a better understanding of the reluctance of complex **5A** to undergo β-H elimination, the analogous gold(iii) *n*-butyl complex **6-Cl** was synthesized and the behaviour of its cationic version was investigated (Scheme 6). The selective mono-alkylation of [(N,C)Au(OAc^F^)_2_] complex **2** was successfully accomplished using 2.1 equiv. of the Grignard reagent *n*BuMgCl under identical conditions to those applied for the preparation of the [(N,C)AuBrPh] complex **3-Br**. After column chromatography, the target [(N,C)AuCl(*n*Bu)] **6-Cl** complex was isolated as a bench-top stable white powder in 73% yield and characterized by multinuclear NMR spectroscopy and high-resolution mass spectrometry.^18^

We then proceeded with the generation of the corresponding cationic gold(iii) complex **6A** by chloride abstraction with AgSbF_6_ in CD_2_Cl_2_. As indicated by ^1^H NMR, it is instantaneously formed at –60 °C and it remains unchanged upon warming up to room temperature. No signs of degradation or β-H elimination (formation of butenes and butane) were observed by ^1^H and ^13^C NMR, even after heating at 40 °C for 24 h. In consequence, the cationic complex was fully characterized by NMR spectroscopy at room temperature without particular precautions. The ^13^C NMR data reveal a significant shift to high field of the aromatic carbon directly bound to gold upon cationization (from *δ* 145.37 ppm in the neutral complex **6-Cl** to *δ* 133.76 ppm in the cationic complex **6A**). On the other hand, the alkylic carbon of the *n*-butyl chain attached to gold shifts to low field with Δ*δ* = 4.6 ppm. Overall, the salient thermal stability of the *n*-butyl cationic complex is in accordance with the lack of β-H elimination in the ethylene insertion reaction of [(N,C)AuPh]^+^ complex **5A**.

In order to explain the double insertion of ethylene and selective formation of **5A**, and to understand the different outcomes observed with the (N,C) and (P,C) complexes, a thorough DFT study was performed.

### Computational investigation of the energy profile for the reaction of **1A** with ethylene

**Fig. 1:**
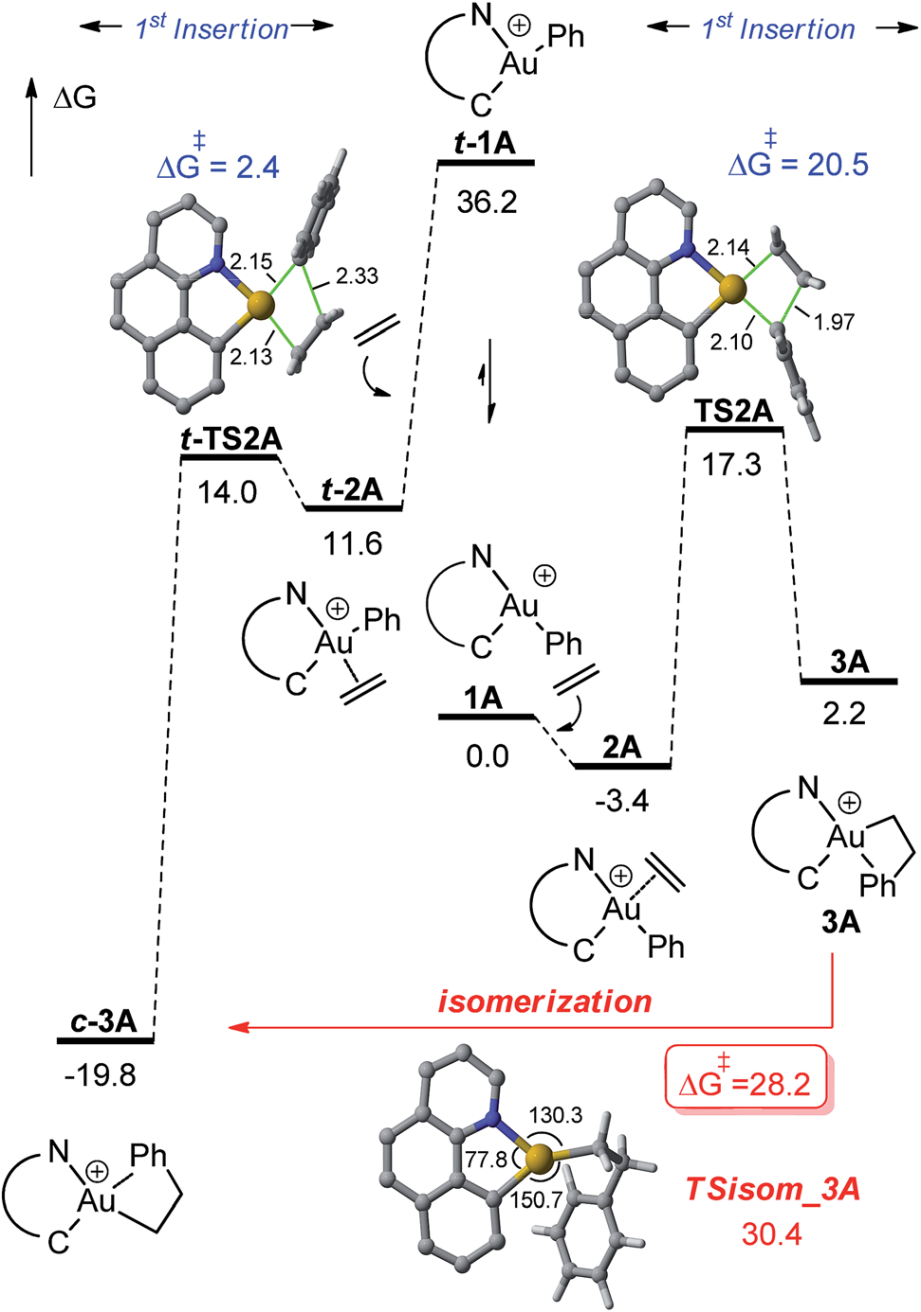
Energy profile (Δ*G* in kcal mol^–1^) computed at the PCM(dichloromethane)-B3PW91/SDD + f(Au)/6-31G**(other atoms) level of theory for the insertion of ethylene into the Au–Ph bond of **1A** (*cis* and *trans* isomers).

DFT calculations were carried out at the B3PW91/SDD + f(Au),6-31G**(other atoms) level of theory. Solvent effects (DCM) were taken into account by means of a polarizable continuum model (PCM).^18^ Two energy minima corresponding to the *cis* and *trans* isomers of **1A** were found on the potential energy surface (PES) (Fig. 1). By convention, *cis* and *trans* refer to the relative position of the phenyl (or alkyl group) at gold with respect to the carbon atom of the benzoquinolinic ligand. The *cis* isomer **1A** is much more thermodynamically stable (by 36.2 kcal mol^–1^), in line with the detrimental *trans* arrangement of the phenyl and benzoquinolinic carbon atoms in ***t*-1A**.

#### 1. First ethylene insertion

Compared to **1A** in its ground-state *cis* geometry, the π-complex of ethylene **2A** is slightly downhill in energy (Δ*G* –3.4 kcal mol^–1^) (Fig. 1). The alkene is coordinated in a symmetric fashion (two short Au···C contacts at 2.40 Å). It is oriented perpendicular to the metal coordination plane16c,^19^,^20^ and the CH_2_ moiety atoms remain quasi-planar, in line with strong π(CC) → Au donation and weak Au → π*(CC) backdonation, as apparent from the NBO second order perturbation analysis (stabilization energies for the donor–acceptor interactions of 45.7 and 14.1 kcal mol^–1^, respectively). Insertion of ethylene into the Au–Ph bond then proceeds *via* a planar 4-center transition state. The resulting Au complex **3A** is stabilized by π–arene coordination *trans* to the weak N donor (Au···C_ortho_ and Au···C_ipso_ contacts of 2.21 and 2.71 Å, respectively and NBO second order stabilization energies for the π(C_ipso_C_ortho_) → σ*(AuN) donor–acceptor interaction of 31.0 kcal mol^–1^).^21^ The activation barrier for the migratory insertion is 20.7 kcal mol^–1^ and the reaction is endergonic (Δ*G* +5.6 kcal mol^–1^). The involvement of the *trans* isomer ***t-*1A** was also considered, but as mentioned above, it lies very high in energy (36.2 kcal mol^–1^ above the *cis* isomer) and is thus very unlikely to participate. Note however that the very unfavourable *trans* arrangement of the two organic fragments makes the coordination–insertion of ethylene into ***t-*1A** very easy and strongly exergonic. Although the resulting insertion product **c-3A** is more stable than **3A** by 22.0 kcal mol^–1^, its involvement is unlikely because the barrier for *trans* → *cis* isomerization is also very high (28.2 kcal mol^–1^) due to the necessity to break the strong π–arene interaction.

#### 2. Second ethylene insertion *versus* β-H elimination

**Fig. 2:**
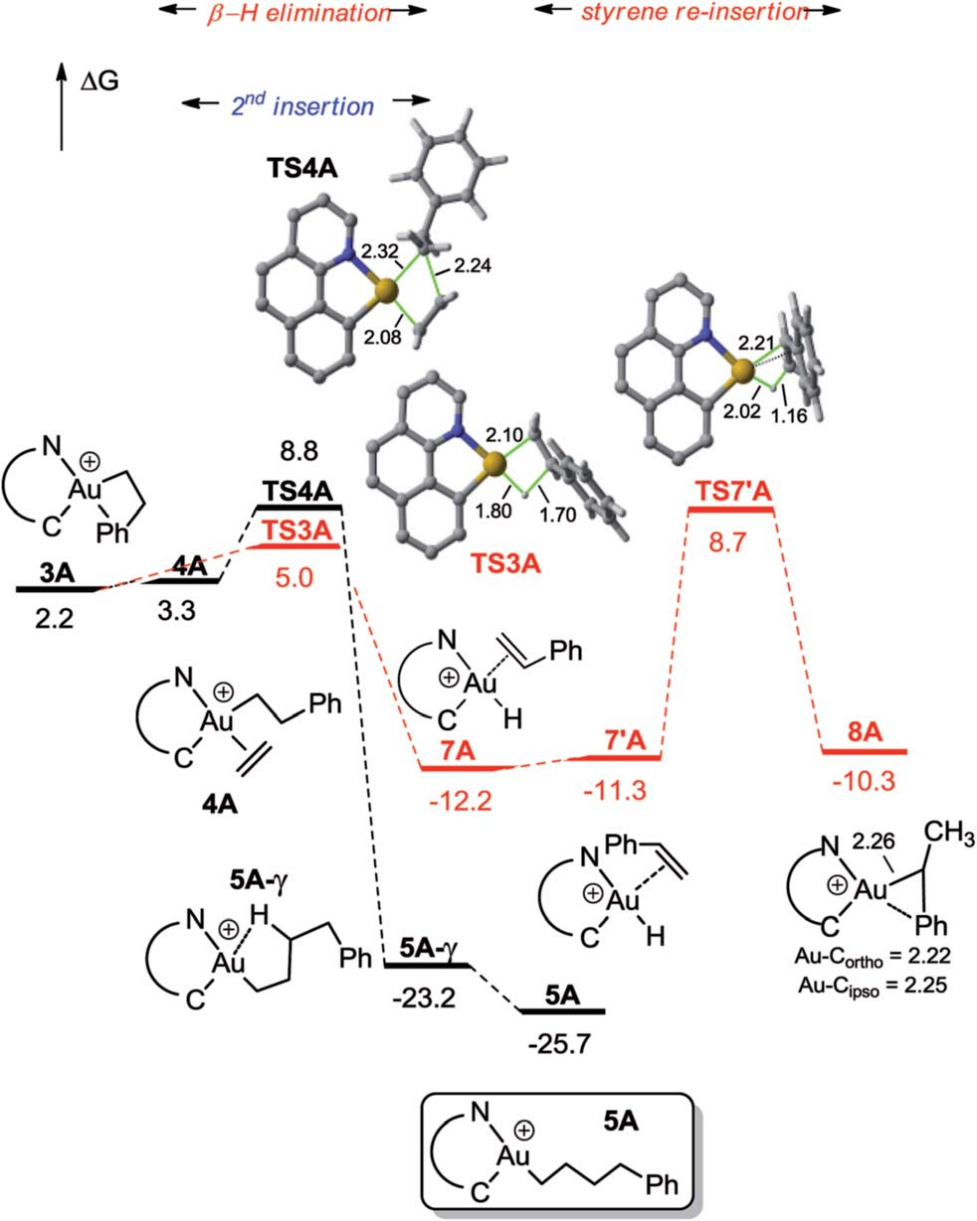
Energy profile (Δ*G* in kcal mol^–1^) computed at the PCM(dichloromethane)-B3PW91/SDD + f(Au)/6-31G**(other atoms) level of theory for the second ethylene insertion and β-H elimination from **3A**.

In line with experimental observations, the insertion of a second ethylene molecule is highly favoured. Displacement of the π–arene coordination at **3A** by ethylene is about thermoneutral and migratory insertion then proceeds with a very low energy barrier (6.6 kcal mol^–1^ for **3A**) to give the (CH_2_)_4_Ph complex **5A** (Fig. 2). The extended linear form **5A** is slightly more stable (by 2.5 kcal mol^–1^) than the γ-CH agostic stabilized one **5A-γ** [*a* 7.1 kcal mol^–1^ σ(CH) → σ*(AuC) donor–acceptor interaction is found at the second-order perturbation level in NBO].^18^,^22^ β-H elimination from **3A** was also considered. In line with that observed for [(P,C)Au(*n*-alkyl)]^+^ complexes, the activation barrier for β-H elimination is low (only 2.8 kcal mol^–1^) and because of the unfavourable *trans* arrangement of **3A**, the formation of the gold hydride styrene complex **7A** is thermodynamically favoured (by 14.4 kcal mol^–1^, Δ*G*). Rotation of styrene and re-insertion into the Au–H bond may occur to give the branched complex **8A** as in the case of the (P,C) complex, but the corresponding energy barrier is relatively high (20.9 kcal mol^–1^). From a kinetic viewpoint, the second ethylene insertion and β-H elimination from **3A** are likely to compete, but the absence of noticeable H/D scrambling upon reaction with ethylene-*d*_2_ (Scheme 5) indicates a minimal occurrence, if any, of β-H elimination. The easiness of the re-insertion of styrene into **7A** (Δ*G*^≠^ 17.2 kcal mol^–1^) combined with the high exergonic character of the second ethylene insertion into **3A** drives the reaction to the formation of **5A**.

For the sake of completeness, β-H elimination, γ-H transfer and third ethylene insertion into **5A**, as well as second ethylene insertion into **8A** have been considered but all these transformations were found to be kinetically or thermodynamically disfavoured (Fig. S7 and S9–S11†).^18^ The same holds true for the second ethylene insertion and β-H elimination from ***c*-3A**, the more stable isomer of the first ethylene insertion. It is in particular worth noting that if the activation barrier for β-H elimination from **5A** is not prohibitively high (19.0 kcal mol^–1^), the reaction is strongly endergonic (Δ*G* 18.5 kcal mol^–1^) due to the unfavourable *trans* arrangement of the resulting gold hydride olefin complex. This probably explains the stability of the [(N,C)Au(*n*Bu)]^+^ complex **6A** noticed experimentally. The activation barrier for the third ethylene insertion is *ca.* 17 kcal mol^–1^ higher than that of the first insertion, in line with the higher reactivity of the Au–C_sp^2^_*vs.* Au–C_sp^3^_ bond, as previously observed with the [(P,C)Ph]^+^ and [(P,C)AuMe]^+^ complexes.16d,e

### Comparison of the (N,C) and (P,C) complexes

The above discussion has pointed out noticeable differences between the (N,C) and (P,C)-ligated gold complexes which are mainly associated with the weaker donor character of N *versus* P. As a result, the (N,C) ligand induces significant electronic dissymmetry on gold compared to the (P,C) ligand, and the two reaction sites *trans* to N or C (*cf.* square-planar geometry of the Au(iii) complexes) behave quite differently, as previously noticed by Tilset, Nova and co-workers for the tolylpyridine (tpy) ligand.13c,d

**Fig. 3:**
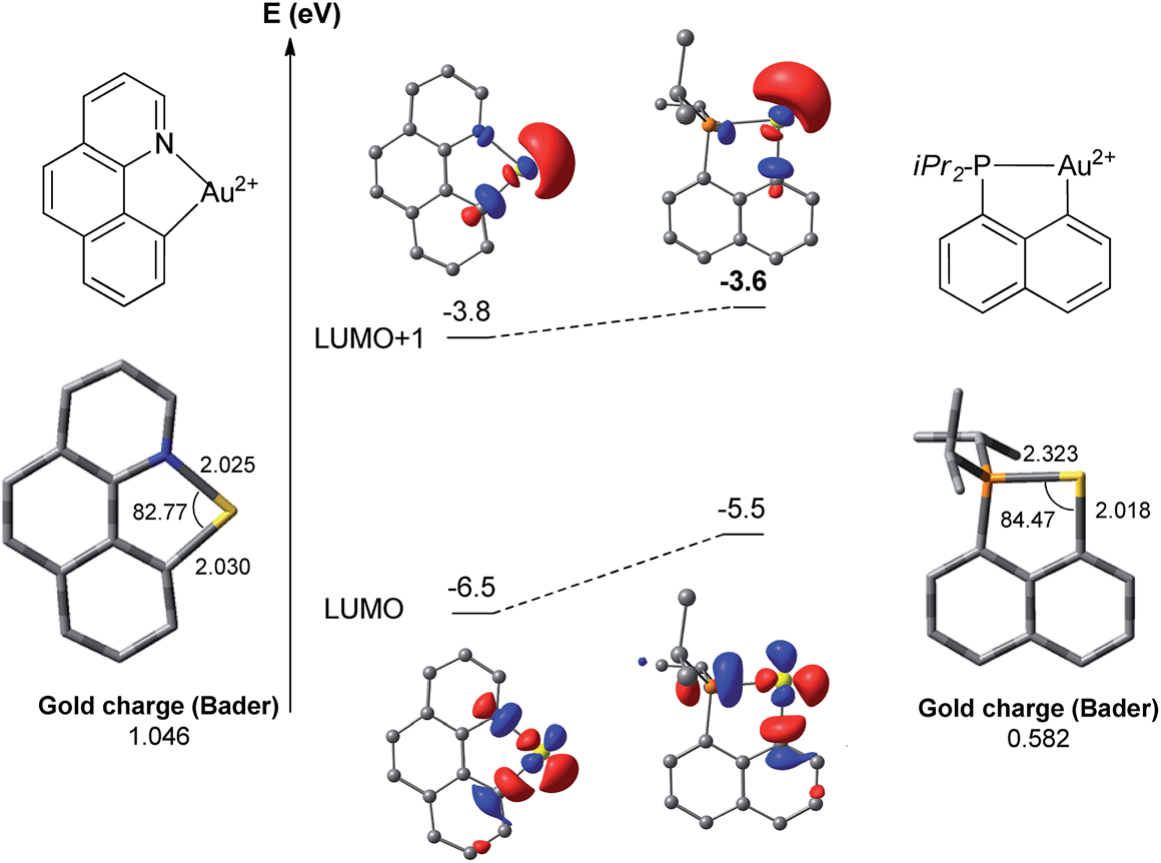
Optimized structures (with selected geometrical data) and the lowest energy molecular orbitals of the [(N,C)Au]^2+^ and [(P,C)Au]^2+^ fragments computed at the PCM(dichloromethane)-B3PW91/SDD + f(Au)/6-31G**(other atoms) level of theory.

To confirm and highlight the difference in the electronic properties of the ligand, calculations were performed on the naked [(N,C)Au]^2+^ and [(P,C)Au]^2+^ fragments (Fig. 3).^18^ In both cases, the LUMO corresponds to the vacant orbital at Au at the *trans* position to the N or P [main contribution of the σ*(Au–N)/(Au–P) orbitals]. It is significantly lower in energy (by 1 eV) with the (N,C) ligand. In contrast, the LUMO + 1 orbitals of the two fragments, which correspond to the vacant orbitals at Au at the *trans* position to the aryl moiety, lie close in energy. Thus, the (N,C) ligand induces a significantly larger LUMO–LUMO + 1 gap than the (P,C) ligand and hence a stronger electronic dissymmetry of the coordination sphere at gold. Note also that the weaker donicity of the (N,C) ligand results in a significantly more electrophilic gold center than with the (P,C) ligand: the LUMO is noticeably lower in energy and the Bader atomic charge at gold is much higher (1.05 *versus* 0.58).

To further compare the two systems and pinpoint the differences, calculations on the [(P,C)AuPh]^+^ complex **1C** were re-performed16*e* at the same level of theory as for the [(N,C)AuPh]^+^ complex **1A** and completed to include the second ethylene insertion (Fig. S12†).^18^ The difference between the (N,C) and (P,C) ligands is spectacular in the energy gap between the *cis* and *trans* isomers of **1A** and **1C** (as well as of related species). As mentioned above, the *cis* form of **1A** is more stable than the *trans* isomer by 36.2 kcal mol^–1^ (Δ*G*). The energy gap falls down to 7.1 kcal mol^–1^ for **1C**. The difference is also striking in the first ethylene insertion as well as in the competition between second ethylene insertion and β-H elimination. With the (N,C) ligand, the first ethylene insertion is slightly uphill in energy (Δ*G* +2.2 kcal mol^–1^) while the second insertion is strongly exergonic (Δ*G* –27.9 kcal mol^–1^) and favoured over β-H elimination. In contrast, ethylene insertion into the (P and C) complex **1C** is downhill in energy (Δ*G* –10.0 kcal mol^–1^) but the second ethylene insertion involves a high activation barrier (21.6 kcal mol^–1^*versus* 15.1 kcal mol^–1^ for the first insertion) and does not compete with β-H elimination/re-insertion leading to the branched product **II**. In addition, reductive elimination of the gold hydride **7A** (C_sp^2^_–H coupling) was found to be significantly more demanding energetically than that of the corresponding (P,C) complex **7C** (the activation barrier is about 10 kcal mol^–1^ higher in energy, see Fig. S14†),^18^ in line with the stability of **5A** and fast decomposition of **II** (see Scheme 4).

## Conclusions

To sum up, the novel cyclometalated Au(iii) complex [(N,C)AuPh]^+^**1A** was shown to react with ethylene to selectively give a double insertion product (**5A**), without β-H elimination nor rearrangement of the linear (CH_2_)_4_Ph chain. Trapping with chloride affords complex **5A-Cl** which has been fully characterized, including by X-ray diffraction. The energy profile for the reactions of **1A** with ethylene has been thoroughly investigated computationally, considering competitive paths, and the influence of the ancillary ligand has been delineated by comparing the (N,C) and (P,C) complexes **1A** and **1C**.

Because of the weak donor character of nitrogen (compared with carbon and even phosphorus), the benzoquinoline ligand is very electronically dissymmetric. As a result, the two reactive sites at gold are quite different. This situation contrasts with the more symmetric nature of the (P,C) chelate and explains the peculiar reactivity of **1A**. The electronic dissymmetry of the (N,C) ligand explains why β-H elimination is not observed upon reaction of **1A** with ethylene, neither upon cationisation of **6-Cl**. The activation barrier for β-H elimination is not prohibitively high, but the reaction is uphill in energy due to the unfavourable position of the hydride (*trans* to the carbon atom of the ligand) in the resulting complex.

The detailed mechanistic study of migratory insertion and β-H elimination has revealed that the properties of the ancillary ligand, and in particular the electronic dissymmetry that the (N,C) ligand induces at gold play a prominent role in reactivity. This situation is somewhat reminiscent of that arising with phosphine–sulfonate bidentate ligands which, thanks to their strong electronic dissymmetry, bestow unique properties to Pd(ii) complexes towards the coordination–insertion polymerization of ethylene and polar olefins.^23^

This study reveals that (N,C) Au(iii) complexes possess rich reactivity. It generalizes migratory insertion of alkenes at gold, an elementary process unknown with gold until very recently, but involved in many synthetically useful transformations (such as the Mizoroki–Heck reaction, the oligomerization/polymerization of olefins, *etc*.). The ancillary ligand markedly influences the outcome of the reaction and thus, modulation of its structure paves the way for tuning and optimizing the properties of gold(iii) complexes.

Accordingly, future work will seek to take advantage of well-defined (N,C) and (P,C) gold(iii) complexes in catalysis and to expand further the variety of chelating ligands.

## Conflicts of interest

There are no conflicts to declare.

## Supplementary Material

Supplementary information

Supplementary information

Crystal structure data
