# Supplementary material for: Cyclometalated gold(iii) complexes: noticeable differences between (N,C) and (P,C) ligands in migratory insertion
Source: Chem Sci. 2018 Mar 26;9(16):3932–40. doi: 10.1039/c7sc04899h (PMC5941201; doi:10.1039/c7sc04899h)

Electronic Supplementary Material (ESI) for Chemical Science.
This journal is © The Royal Society of Chemistry 2018

Supporting Information

TABLE OF CONTENTS

1. Materials and Methods…...…………………………………………………………………..S2

1.1 Materials and instrumentation………………………………………………….……...S2

1.2 Synthesis and characterization of the Au(III) complexes…… ………………………....S2

2. Computational Details…………………………………………………………………..S6

3. Figures and Tables....……………………………………………………………………S8

4. X-ray Crystallography Data………………………………………………… …………..S32

1. Materials and Methods

1.1. Materials and instrumentation

Reagents and solvents used were commercially available reagent quality unless indicated otherwise. *Trans*-ethylene-d_2 was purchased from Cluzeau Info Labo and supplied in a 1 atm lecture bottle (1 atm, 95.2% D). All reactions and manipulations were carried out under an atmosphere of dry argon using standard Schlenk techniques or in a glovebox under inert atmosphere with O_2 and H_2O concentrations < 1.0 ppm. Dry, oxygen-free solvents were employed. Solution ^{1}H, ^{13}C and ^{19}F NMR spectra were recorded on Bruker AVANCE 300, 400 or 500 spectrometers under routine conditions at 298 K unless otherwise specified. The following abbreviations and their combinations are used: br, broad; s, singlet; d, doublet; t, triplet; q, quartet, m, multiplet. The ^{1}H and ^{13}C resonance signals were attributed by means of 2D COSY, HSQC and HMBC experiments. High resolution mass spectra (HRMS) were recorded on a Waters LCT apparatus and/or a Bruker MicrOTOF-Q II TM instrument using ESI or Cryospray ionization sources.

1.2. Synthesis and characterization of the Au(III) complexes

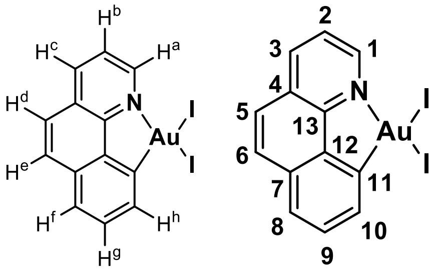

Complex **1** was prepared following a previously described procedure.[1]To a mixture of gold iodide (194.9 mg, 0.6 mmol) and 10-Iodobenzo[*h*]quinolone **2a-I**(183.7 mg, 0.6 mmol) was added toluene (4 mL) at room temperature and stirred at 60°C for 18h. The resulting suspension was then dried under vacuum and washed with cold DCM to yield **1** as a red powder (362.3 mg, 96%).**^{1}H-NMR** ($CDCl_3$, 400 MHz, 323K) δ, ppm: 10.36 (dd, *J* = 5.6, 1.2 Hz, 1H, H^a), 8.93 (dd, *J* = 8.0, 0.8 Hz, 1H, H^h), 8.55 (dd, *J* = 8.0, 0.4 Hz, 1H, H^c), 7.88 (d, *J* = 8.8 Hz, 1H, H^e), 7.87 (d, *J* = 8.0 Hz, 1H, H^f), 7.79 (dd, *J* = 8.0, 5.6 Hz, 1H, H^b), 7.71 (d, *J* = 8.7 Hz, 1H, H^d), 7.59 (t, *J* = 8.0 Hz, 1H, H^g); **^{13}C{1H}-NMR** ($CDCl_3$, 100 MHz, 323K) δ, ppm: 154.81 (C13), 153.63 (C11), 150.00 (C1), 140.64 (C12), 140.26 (C3), 135.88 (C7), 134.52 (C10), 131.44 (C9), 130.38 (C6), 129.22 (C4), 126.33 (C8), 124.06 (C5), 122.60 (C2).

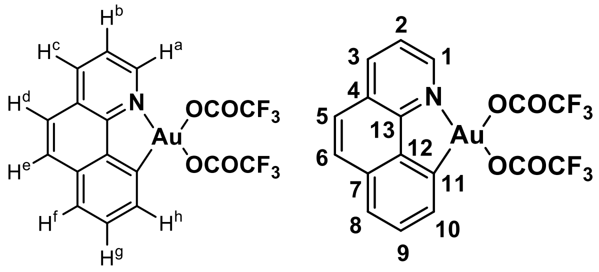

2: Complex**1** (248.1 mg, 0.39mmol) and $AgOAc^F$ (183.3 mg, 0.83mmol) were suspended in CH_2Cl_2 (8 mL). After stirring for 10 min at room temperature, solvent was removed under vacuum and acetone (8 mL) was added. Theresulting light yellow mixture was filtered through *Celite*®, dried under vacuum and washed with hexanes (3 x 3 mL) to give the

[1] J. Serra, T. Parella, X. Ribas, *Chem. Sci*. **2017**, *8*, 946.

desired complex as a pale yellow solid (213.4 mg, 91%).**^{1}H-NMR** (acetone-d_6,400 MHz, 298K) δ, ppm: 9.08 (dd,*J* = 8.0, 1.2 Hz, 1H, H^{c}), 8.95(dd, *J* = 5.6, 1.2 Hz, 1H, H^{a}), 8.16 (dd, *J* = 8.0, 5.6 Hz, 1H, H^{b}), 8.10 (m, 3H, H^{d}+H^{e}+H^{f}), 7.72 (t, *J* = 8.0 Hz, 1H, H^{g}), 7.25 (d, *J* = 7.6 Hz, 1H, H^{h}); **^{13}C{1H}-NMR** (acetone-d_6, 100 MHz, 298K) δ, ppm: 153.32 (C13), 148.15(C1), 143.44 (C3), 140.54 (C11), 137.18 (C12), 135.52 (C7), 130.22 (C9), 129.60 (C5), 129.35 (C4), 128.29 (C8), 126.22 (C10), 125.50 (C6), 124.08 (C4); **^{19}F{1H}-NMR** (acetone-d_6, 282 MHz, 298K) δ, ppm: -74.85 (s, CF_3*trans to N*, 3F), -73.99 (s, br, CF_3*cis to N*, 3F); **HRMS (ESI)** (Acetone/CH_3CN, m/z): calcd for $C_{17}H_8AuF_6NO_4$ $[M+Na]^+$623.9915, found: 623.9962.

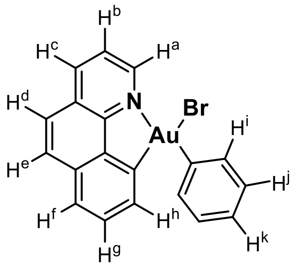

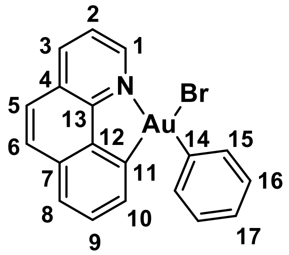

3-Br: To a solution of complex **2**(210.5 mg, 0.35mmol) in THF (10 mL) was added dropwise PhMgBr (2.1 equiv, 2M in diethyl ether) at -78ºC. The reaction mixture was stirred at -78ºC for 1 h and then at room temperature for 1 h. The solvent was removedinvacuo and the resulting solid was dissolved in CH_2Cl_2 (8 mL) and washed with distilled water (2 x 2 mL). The organic phase was dried over Na_2SO_4, filtered through *Celite*® and all volatiles were then removed under vacuum to give a pale yellow solid, which was finally purified by column chromatography on silica gel (CH_2Cl_2:hexane 3:2) to yield**3-Br** as a white solid (110.0 mg, 59%).**^{1}H-NMR** ($CDCl_3$, 500 MHz, 298K) δ, ppm: 9.89 (dd, *J* = 5.0, 1.5 Hz, 1H, H^{a}), 8.53 (dd, *J* = 8.0, 1.5 Hz, 1H, H^{c}), 7.93 (d, *J* = 8.5 Hz, 1H, H^{e}), 7.85 (dd, *J* = 8.0, 5.0 Hz, 1H, H^{b}), 7.83 (dd, *J* = 7.5, 0.5 Hz, 1H, H^{f}), 7.78 (d, *J* = 8.5 Hz, 1H, H^{d}), 7.61 (dd, *J* = 8.0, 1.0 Hz, 2H, H^{i}), 7.54 (t, *J* = 7.5 Hz, 1H, H^{g}), 7.31 (t, *J* = 7.5 Hz, 2H, H^{j}), 7.25 (tt, *J* = 7.5, 1.5 Hz, 1H, H^{k}), 7.06 (dd, *J* = 7.5, 1.0 Hz, 1H, H^{h}); **^{13}C{1H}-NMR** ($CDCl_3$, 125 MHz, 298K) δ, ppm: 151.97 (C13), 149.41 (C11), 148.68 (C1), 141.18 (C14), 139.56 (C3), 138.60 (C7), 134.50 (C12), 133.08 (C15), 131.66 (C10), 130.44 (C9), 129.70 (C6), 129.44 (C16), 127.77 (C4), 126.21 (C8), 125.97 (C17), 124.11 (C5), 122.91 (C2); **HRMS (ESI)** ($CHCl_3$, m/z): calcd for $C_{19}H_{13}AuN$ $[M^+]$ 452.0714, found: 452.0722.**Anal. Calcd for $C_{19}H_{13}AuBrN$**: C, 42.88; H, 2.46; N, 2.63. Found: C, 42.91; H, 2.34; N, 2.98.

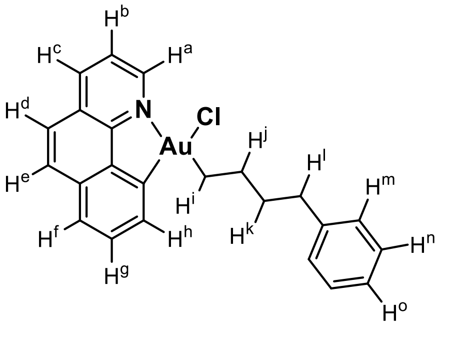

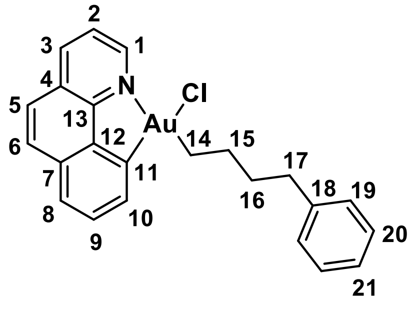

5A-Cl: In an inert-atmosphere glovebox, a pressure screw-cap NMR tube was charged with **3-Br** (16.0 mg, 0.03mmol) in CD_2Cl_2 (0.35 mL). $AgSbF_6$ (12.4 mg, 0.036mmol) was placed into a small glass vial and solubilized in

CD_2Cl_2 (0.35 mL). The solution was loaded into a plastic syringe equipped with a stainless steel needle, and the syringe was closed by blocking the needle with a septum. Outside the glovebox, the NMR tube was pressurized under argon atmosphere and cooled down to –60 °C (Acetone/N_2coldbath). At this temperature, the solution of $AgSbF_6$ was added. The overall mixture was degassed 3 times using the Freeze-Pump-Thaw degassing technique and 2 bar of ethylene were added. The reaction was monitored by ^{1}H-NMR. After heating overnight at 40°C the ethylene pressure was released and the reaction crude was filtered through *Celite*® on a vial-containing *n*Bu_4N-Cl (10.0 mg, 0.036 mmol). The resulting solution was purified by column chromatography on silica gel (CH_2Cl_2:hexane 2:1) to give**5A-Cl** as a white solid (9.6 mg, 60%). **^{1}H-NMR** ($CDCl_3$, 400 MHz, 298K) δ, ppm: 9.58 (dd, *J* = 5.2, 1.6 Hz, 1H, H^a), 8.46 (dd, *J* = 8.0, 1.6 Hz, 1H, H^c), 7.92 (d, *J* = 8.8 Hz, 1H, H^d), 7.86 (dd, *J* = 7.6, 0.8 Hz, 1H, H^f), 7.82 (m, *2*H, H^b + H^h), 7.75 (d, *J* = 8.8 Hz, 1H, H^e), 7.74 (t, *J* = 7.6 Hz, 1H, H^g), 7.26 (m, 4H, H^m + H^n), 7.18 (tt, *J* = 7.2, 1.6 Hz, 1H, H^o), 2.76 (t, *J* = 8.0 Hz, 2H, H^l), 2.72 (t, *J* = 8.0 Hz, 2H, H^i), 1.97 (m, 4H, H^j + H^k); **^{13}C{1H}-NMR** ($CDCl_3$, 100 MHz, 298K) δ, ppm: 150.81 (C13), 147.00 (C1), 145.37 (C11), 142.70 (C18), 139.20 (C7), 139.08 (C3), 134.71 (C12), 130.22 (C9), 129.56 (C5), 128.51 (C19), 128.23 (C20), 128.13 (C10), 127.41 (C4), 126.22 (C8), 125.57 (C21), 124.07 (C6), 122.73 (C2), 35.78 (C17), 34.24 (C14), 34.13 (C15), 30.84 (C16); **HRMS (ESI)** ($CHCl_3$, m/z): calcd for $C_{23}H_{21}AuN$ [M^+] 508.1340, found: 508.1346; **Anal. Calcd for $C_{23}H_{21}AuNCl$**: C, 50.80; H, 3.89; N, 2.58. Found: C, 50.45; H, 3.48; N, 2.89.

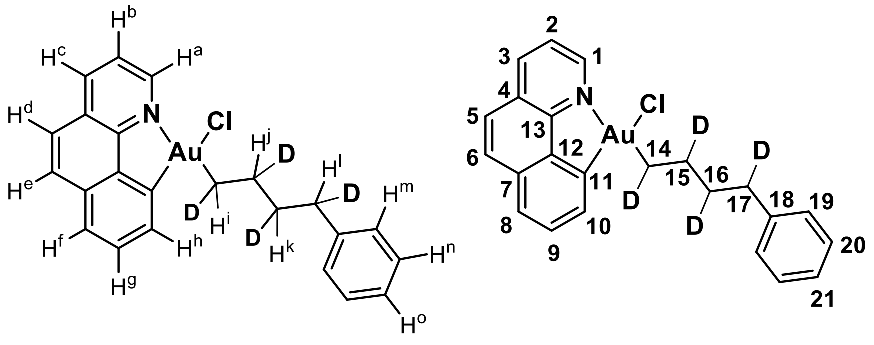

5A-Cl-*d₄*: In an inert-atmosphere glovebox, a pressure screw-cap NMR tube was charged with **3-Br** (14.6 mg, 0.027 mmol) in CD_2Cl_2 (0.3mL). $AgSbF_6$ (12.0mg, 0.035 mmol) was placed into a small glass vial and solubilized in CD_2Cl_2 (0.3 mL). The solution was loaded into a plastic syringe equipped with a stainless steel needle, and the syringe was closed by blocking the needle with a septum. Outside the glovebox, the NMR tube was filled with argon and cooled down to –60 °C (Acetone/N_2 coldbath). At this temperature, the solution of $AgSbF_6$ was added. The overall mixture was degassed 3 times using the Freeze-Pump-Thaw degassing technique and 1 bar of *trans*-ethylene-*d_2* was added. After heating for 24 h at 40°C, the NMR tube was refilled with 1 bar of *trans*-ethylene-*d_2* and the mixture was heated for 24 h more at 40°C. Then, in an inert-atmosphere glovebox, the reaction crude was filtered through *Celite*® on a vial-containing *n*Bu_4NCl (9.2 mg, 0.033 mmol). The resulting solution was analyzed by NMR without further purification. **5A-Cl-*d_4*** was isolated as a white solid (5.0 mg,

33%) by column chromatography on silica gel (CH_2Cl_2:hexane 2:1). **^{1}H-NMR** (CD_2Cl_2, 500 MHz, 298K) δ, ppm: 9.43 (dd, *J* = 5.1, 1.5 Hz, 1H, H^a), 8.45 (dd, *J* = 8.0, 1.5 Hz, 1H, H^c), 7.88 (d, *J* = 8.8 Hz, 1H, H^d), 7.83 (dd, *J* = 7.9, 0.8 Hz, 1H, H^f), 7.80 – 7.67 (m, 4H, H^b + H^h + H^e + H^g), 7.27 – 7.19 (m, 4H, H^m + H^n), 7.15 (tt, *J* = 7.2, 1.6 Hz, 1H, H^o), 2.69 (d, *J* = 6.1 Hz, 1H, H^l), 2.59 (d, *J* = 5.7 Hz, 1H, H^i), 1.88 (t, *J* = 7.0 Hz, 1H, H^j or H^k), 1.84 (t, *J* = 7.0 Hz, 1H, H^j or H^k); **^{13}C{1H}-NMR** (CD_2Cl_2, 125 MHz, 298K) δ, ppm: 151.18 (C13), 147.29 (C1), 145.77 (C11), 143.38 (C18), 139.83 (C3), 139.58 (C7), 135.19 (C12), 130.62 (C9), 129.93 (C5), 128.98 (C19), 128.72 (C20), 128.48 (C10), 127.99 (C4), 126.72 (C8), 126.07 (C21), 124.76 (C6), 123.39 (C2), 35.77 (t, C17), 34.23 (t, C14), 33.67 (t, C15), 30.73 (t, C16); **HRMS (ESI)** ($CHCl_3$, m/z): calcd for $C_{23}H_{17}D_4AuN$ [M^+] 512.1591, found: 512.1559.

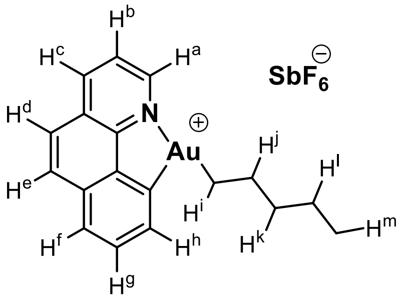

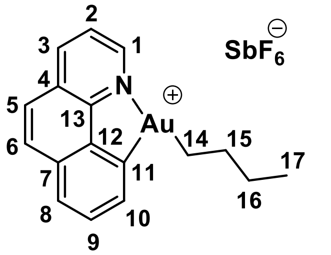

6A: In an inert-atmosphere glovebox, a pressure screw-cap NMR tube was charged with **6-Cl** (9.4 mg, 0.02 mmol) in CD_2Cl_2 (0.35 mL). $AgSbF_6$ (8.2 mg, 0.024 mmol) was placed into a small glass vial and solubilized also in CD_2Cl_2 (0.35 mL). The solution was loaded into a plastic syringe equipped with a stainless steel needle, and the syringe was closed by blocking the needle with a septum. Outside the glovebox, the NMR tube was pressurized under argon atmosphere and cooled down to –60 °C (Acetone/N_2coldbath). At this temperature, the solution of $AgSbF_6$ was added. The tube was immediately introduced in the NMR machine and the reaction was monitored by ^{1}H-NMR from -60°C to room temperature. Complex **6A** was generated at -60°C and no formation of butane or 2-butenes was observed upon warming. The complex was characterized at room temperature: **^{1}H-NMR** (CD_2Cl_2, 400 MHz, 298K) δ, ppm: 8.98 (dd, *J* = 3.6 Hz, 1H, H^a), 8.63 (dd, *J* = 8.0 Hz, 1H, H^c) 8.00 (m, 3H, H^b + H^e + H^f), 7.89 (d, *J* = 8.8 Hz, 1H, H^d), 7.79 (m, 2H, H^h+ H^g), 2.58 (dd, *J* = 7.6 Hz, 2H, H^i), 1.88 (dd, *J* = 7.6 Hz, 2H, H^j), 1.65 (sext, *J* = 7.6 Hz, 2H, H^k), 1.07 (t, *J* = 7.6 Hz, 3H, H^l); **^{13}C{1H}-NMR** (CD_2Cl_2, 100 MHz, 298K) δ, ppm: 147.79 (C13), 146.59 (C1), 140.57 (C3), 135.04 (C7), 133.76 (C11), 131.64 (C12), 130.24 (C9), 129.47 (C6), 128.71 (C10), 128.00 (C8), 125.39 (C4), 124.76 (C5), 123.99 (C2), 38.86 (C14), 32.43 (C15), 25.36 (C16), 13.64 (C17); **HRMS (ESI)** (CH_2Cl_2, m/z): calcd for $C_{17}H_{17}AuN$ [M^+] 432.1021, found: 432.1027.

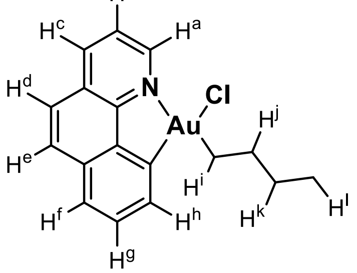

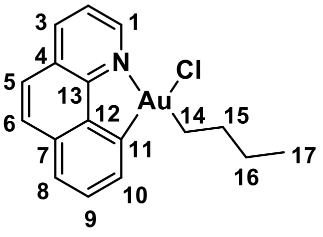

6-Cl: To a solution of complex **2** (41.9 mg, 0.07 mmol) in THF (4 mL) was added dropwise BuMgCl (2.1 equiv, 2M in diethyl ether) at -78°C. The reaction mixture was stirred at -78°C for 1 h and then at room

temperature for 1 h. The solvent was removed in vacuo and the resulting solid was dissolved in CH_2Cl_2 (3 mL) and washed with distilled water (2 x 1 mL). The collected organic phases were dried over Na_2SO_4, filtered through *Celite*® and all volatiles were then removed under vacuum to give the light yellow solid, which was subsequently purified by column chromatography on silica gel (CH_2Cl_2:hexane 3:1) to yield**6-Cl** as a white solid (23.9 mg, 73%).**^{1}H-NMR** ($CDCl_3$, 400 MHz, 298K) δ, ppm: 9.58 (dd, *J* = 5.2, 1.2 Hz, 1H, H^a), 8.46 (dd, *J* = 8.0, 1.2 Hz, 1H, H^c), 7.92 (d, *J* = 8.8 Hz, 1H, H^e), 7.88 (m,2H, H^g + H^f), 7.81 (dd, *J* = 8.0, 5.2 Hz, 1H, H^b), 7.76 (m, 2H, H^h + H^d), 2.71 (t, *J* = 7.6 Hz, 2H, H^i), 1.92 (quint, *J* = 7.6 Hz, 2H, H^j), 1.63 (sext, *J* = 7.6 Hz, 2H, H^k), 1.03 (t, *J* = 7.6 Hz, 3H, H^l); **^{13}C{1H}-NMR** ($CDCl_3$, 100 MHz, 298K) δ, ppm: 150.76 (C13), 146.96 (C1), 145.37 (C11), 139.20 (C7), 139.02 (C3), 134.68 (C4), 130.18 (C10), 129.55 (C6), 128.14 (C9), 127.38 (C12), 126.19 (C8), 124.05 (C5), 122.71 (C2), 34.71 (C14), 33.40 (C15), 25.68 (C16), 14.06 (C17); **HRMS (ESI)** ($CHCl_3$, m/z): calcd for $C_{17}H_{17}AuN$ [M^+] 432.1021, found: 432.1018.

2. Computational Details

All calculations were performed using the Gaussian 09 package[2] and the B3PW91 hybrid[3] functional on the real cationic experimental systems, taking into account solvent effect (dichloromethane) by means of polarizable continuum model PCM.[4] The weakly coordinating counter–anion SbF_6^- has not been considered in the calculations since we previously showed that even a more coordinating counter-anion like NTf_2^- has no significant impact on the reaction profile (migratory insertion, β-hydride elimination) in similar processes than those described in this work.[5] The gold atom was described with the relativistic electron core potential SDD and associated basis set,[6] augmented by a set of f-orbital polarization functions.[7] The 6-31G** basis set were employed for other atoms. All stationary points involved were fully optimized in solvent.

---

[2] Gaussian 09, Revision C.01, M. J. Frisch, G. W. Trucks, H. B. Schlegel, G. E. Scuseria, M. A. Robb, J. R. Cheeseman, G. Scalmani, V. Barone, B. Mennucci, G. A. Petersson, H. Nakatsuji, M. Caricato, X. Li, H. P. Hratchian, A. F. Izmaylov, J. Bloino, G. Zheng, J. L. Sonnenberg, M. Hada, M. Ehara, K. Toyota, R. Fukuda, J. Hasegawa, M. Ishida, T. Nakajima, Y. Honda, O. Kitao, H. Nakai, T. Vreven, J. A. Montgomery, Jr., J. E. Peralta, F. Ogliaro, M. Bearpark, J. J. Heyd, E. Brothers, K. N. Kudin, V. N. Staroverov, T. Keith, R. Kobayashi, J. Normand, K. Raghavachari, A. Rendell, J. C. Burant, S. S. Iyengar, J. Tomasi, M. Cossi, N. Rega, J. M. Millam, M. Klene, J. E. Knox, J. B. Cross, V. Bakken, C. Adamo, J. Jaramillo, R. Gomperts, R. E. Stratmann, O. Yazyev, A. J. Austin, R. Cammi, C. Pomelli, J. W. Ochterski, R. L. Martin, K. Morokuma, V. G. Zakrzewski, G. A. Voth, P. Salvador, J. J. Dannenberg, S. Dapprich, A. D. Daniels, O. Farkas, J. B. Foresman, J. V. Ortiz, J. Cioslowski, and D. J. Fox, Gaussian, Inc., Wallingford CT, **2009**.

[3] (a) A. D. Becke *J. Chem. Phys.***1993**, *98*, 5648; (b) J. P. Perdew, in *Electronic Structure of Solids '91*, Ed. P. Ziesche and H. Eschrig, Akademie Verlag, Berlin, **1991**, 11.

[4] J. Tomasi, B. Mennucci, R. Cammi, *Chem. Rev.***2005**, *105*, 2999.

[5]F. Rekhroukh,L. Estévez,S. Mallet-Ladeira,K. Miqueu,A. Amgoune,D. Bourissou, *J. Am. Chem. Soc.***2016**, *138*,11920.

[6]D.Andrae, U. Häussermann, M. Dolg, H. Stoll, H. Preuss, *Theor. Chim. Acta***1990**, *77*, 123.

[7] A. W. Ehlers, M. Biihme, S. Dapprich, A. Gobbi, A. Hijllwarth, V. Jonas, K. F. Kiihler, R. Stegmann, A. Veldkamp, G. Frenking, *Chem. Phys. Letters***1993**, *208*, 111.

Frequency calculations were undertaken to confirm the nature of the stationary points, yielding one imaginary frequency for transition states (TS), corresponding to the expected process, and all of them positive for *minima*. The connectivity of the transition states and their adjacent *minima* was confirmed by intrinsic reaction coordinate (IRC)[8] calculations.

Natural Bond Orbital[9] calculations (NBO, 5.9 version)[10] have been carried to analyze the bonding situation, in particular for the description of gold π-complexes and complexes involving β-H and γ-H interactions. Natural Localized Molecular Orbital (NLMO) were plotted with Molekel 4.3[11] and all the geometrical structures with Gaussview 5.0[12]and CYLview.[13]

^{13}C NMR chemical shifts for complex **4** (*cis* and *trans* isomers) were evaluated by employing the direct implementation of the Gauge Including Atomic Orbitals (GIAO),[14] with the IGLOII[15] basis set on C, H and N atoms, using as reference the corresponding $SiMe_4$ shielding constant calculated at the same level of theory.

Bader[16] charges have been computed for the dicationicspecies $[(N,C)Au]^{2+}$ and $[(P,C)Au]^{2+}$in order to analyze atomic charge at gold.

---

8 (a) K. Fukui, *Acc. Chem. Res.*, **1981**, *14*,363; (b) H. P. Hratchian, H. B. Schlegel, in *Theory and Applications of Computational Chemistry: The First 40 Years*, Ed. C. E. Dykstra, G. Frenking, K. S. Kim, G. Scuseria, Elsevier, Amsterdam, **2005**, 195.

9(a) E. Reed, L. A. Curtiss, F. Weinhold, *Chem. Rev.* **1988**, *88*, 899; (b) J. P. Foster F. Weinhold, *J. Am. Chem. Soc.***1980**, *102*, 7211; (c) A. E. Reed, F. Weinhold, *J. Chem. Phys.***1985**, *83*, 1736.

10NBO 5.0 program, E. D. Glendening, J, K. Badenhoop, A. E. Reed, J. E. Carpenter, J. A. Bohmann, C. M. Morales, F. Weinhold, Theoretical Chemistry Institute, University of Wisconsin, Madison, **2001**.

11 MOLEKEL 4.3, P. Flükiger, H. P. Lüthi, S. Portmann, J. Weber, Swiss Center for Scientific Computing, Manno (Switzerland), **2000-2002**.

12 GaussView, Version 5, R. Dennington, T. Keith, J. Millam, *Semichem Inc.*, Shawnee Mission, KS, **2009**.

13 C.Y. Legault, CYLview, 1.0b, Université de Sherbrooke, **2009** (http://www.cylview.org).

14 (a) F. London, *J. Phys. Radium***1937**, *8*, 397; (b) R. McWeeny, *Phys. Rev.***1962**, *126*, 1028; (c) R. Ditchfield, *Mol. Phys.***1974**, *27*, 789; (d) K. Wolinski, J. F. Hilton, P. Pulay, *J. Am. Chem. Soc.***1990**, *112*, 8251; (e) J. R. Cheeseman, G. W. Trucks, T. A. Keith, M. J. Frisch, *J. Chem. Phys.***1996**, *104*, 5497.

15 W. Kutzelnigg, U. Fleischer, M. Schindler, *The IGLO-Method: Ab Initio Calculation and Interpretation of NMR Chemical Shifts and Magnetic Susceptibilities*, Springer-Verlag, Heidelberg, **1990**, vol. 23.

16 (a) R. F. W. Bader, *Atoms in Molecules: A Quantum Theory*; Oxford University Press: New-York Ed., **1990**; (b) R. F. W. Bader, *Chem. Rev.***1991**, *91*, 893; (c) AIMAll (Version 10.10.11), Todd A. Keith, **2010** (aim.tkgristmill.com).

3. Figures and Tables

Figure S1. a) ^{1}H-NMR spectrum of complex **2** inacetone-d_6, 400 MHz, 298 K; b) ^{13}C{^{1}H}-NMR spectrum (acetone-d_6, 100 MHz, 298 K); c) ^{19}F{^{1}H}-NMR spectrum (acetone-d_6, 282 MHz, 298 K).

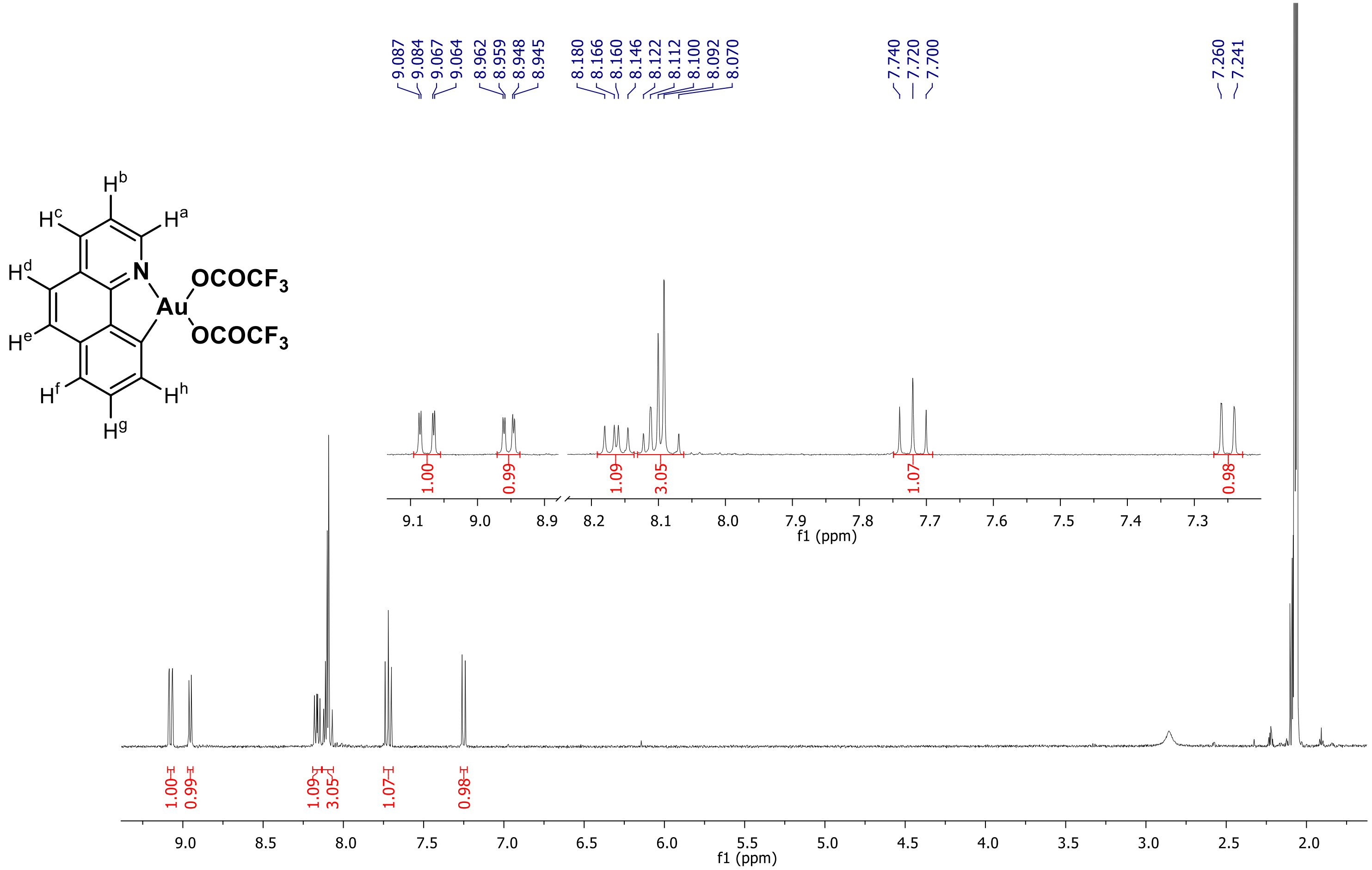

b)

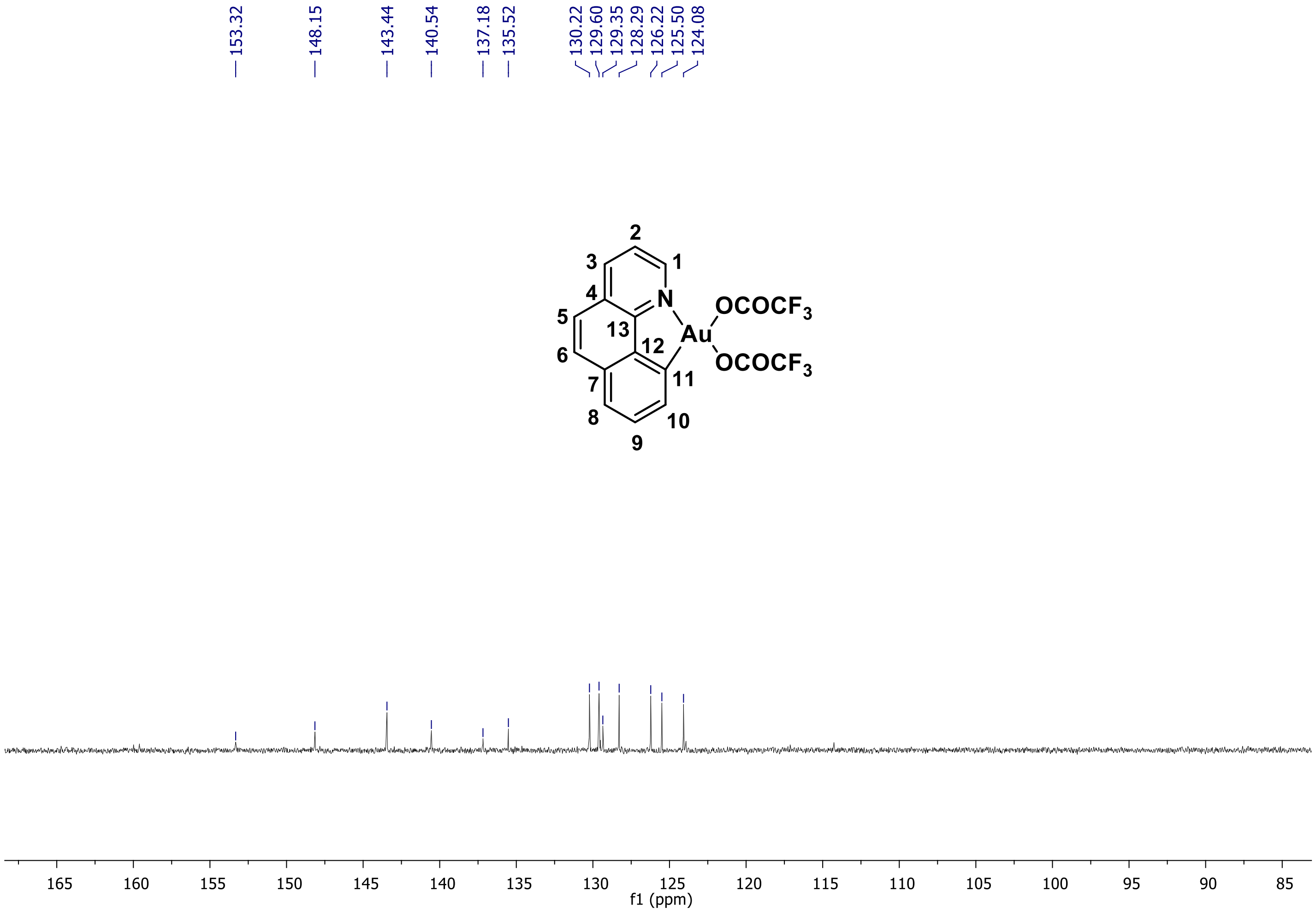
153.32
148.15
143.44
140.54
137.18
135.52
130.22
129.60
129.35
128.29
126.22
125.50
124.08
1
2
3
4
5
6
7
8
9
10
11
12
13
N
Au
OCOCF3
OCOCF3
165 160 155 150 145 140 135 130 125 120 115 110 105 100 95 90 85
f1 (ppm)

c)

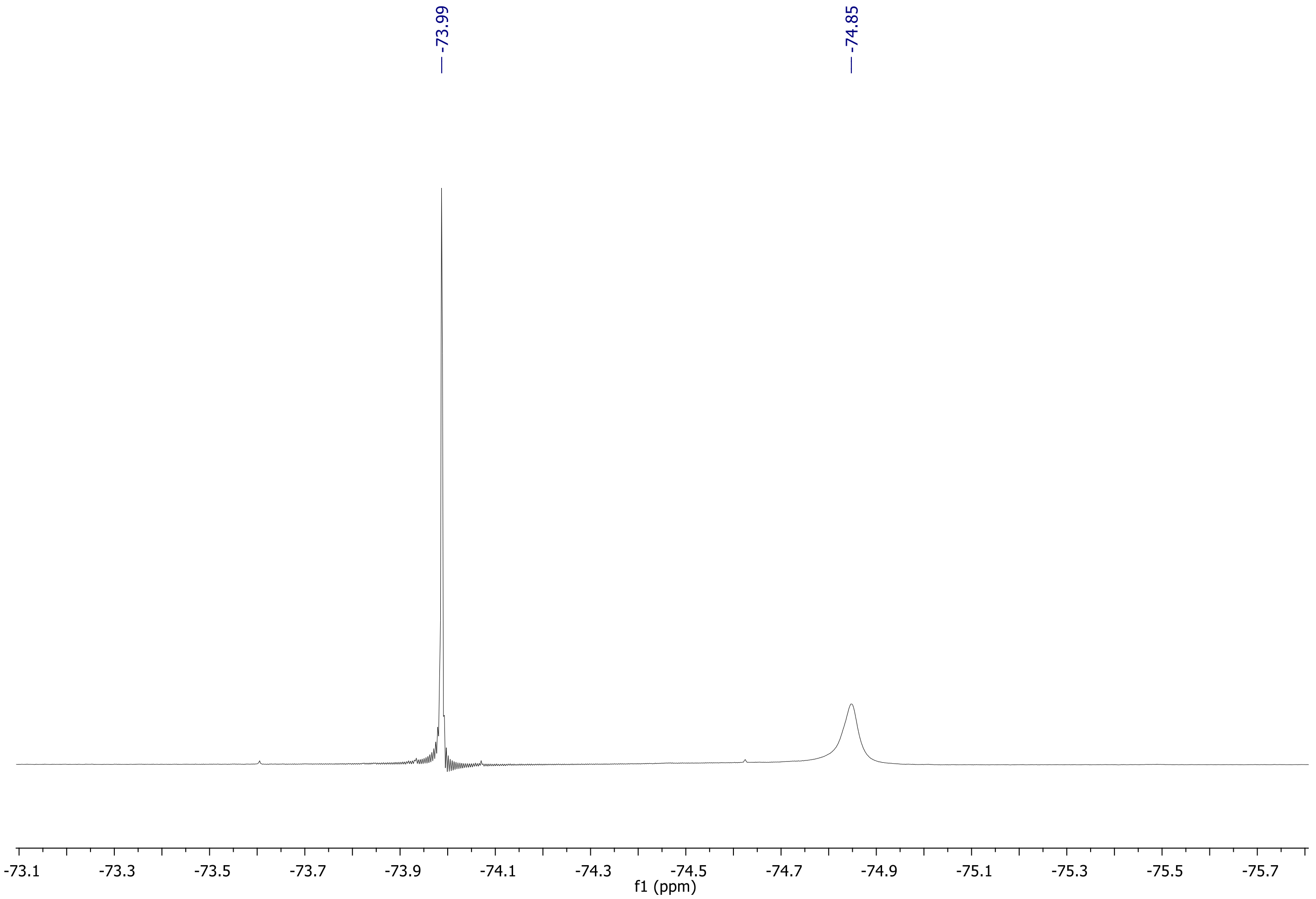

-73.99
-74.85
-73.1
-73.3
-73.5
-73.7
-73.9
-74.1
-74.3
-74.5
-74.7
-74.9
-75.1
-75.3
-75.5
-75.7
f1 (ppm)

Figure S2. a) ^{1}H-NMR spectrum of complex **3-Br** in $CDCl_3$, 500 MHz, at 298 K; b) $^{13}C\{^{1}H\}$-NMR spectrum ($CDCl_3$, 125 MHz, 298 K).

a)

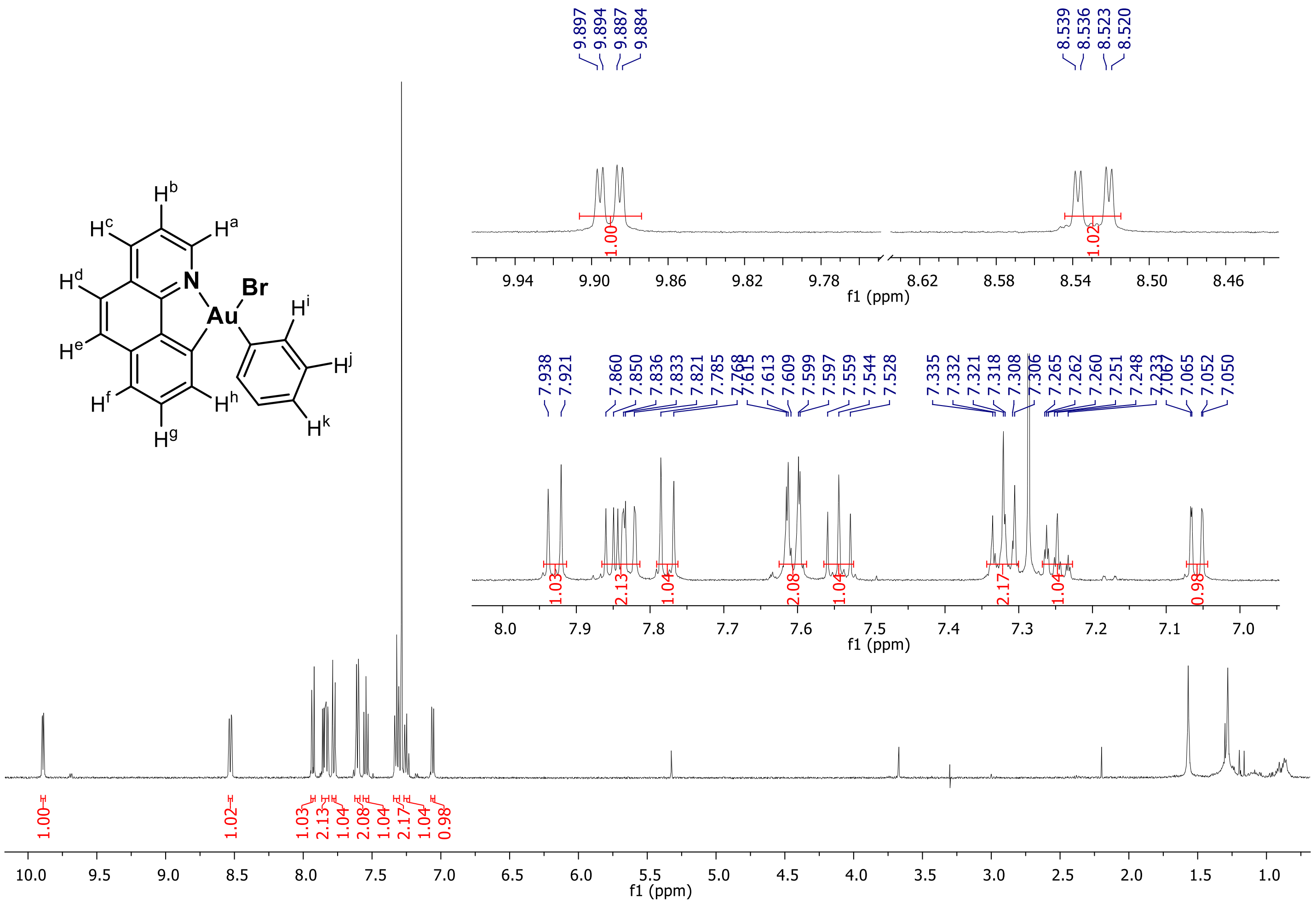

b)

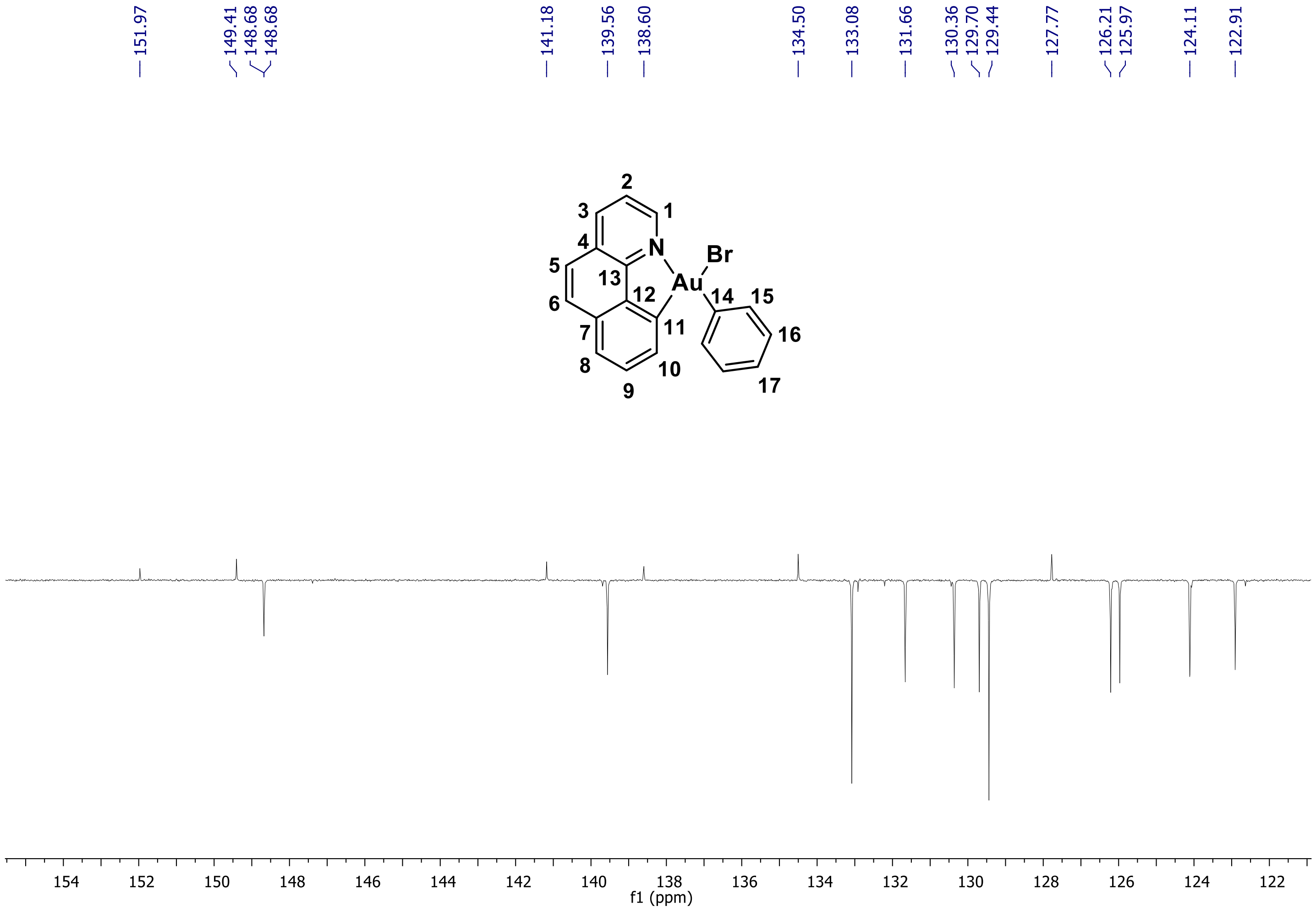
151.97
149.41
148.68
148.68
141.18
139.56
138.60
134.50
133.08
131.66
130.36
129.70
129.44
127.77
126.21
125.97
124.11
122.91
Br
Au
N
1
2
3
4
5
6
7
8
9
10
11
12
13
14
15
16
17
154
152
150
148
146
144
142
140
138
136
134
132
130
128
126
124
122
f1 (ppm)

Figure S3. a) ^{1}H-NMR spectrum of complex **5A-Cl** in $CDCl_3$, 400 MHz, at 298 K; b) $^{13}C\{^{1}H\}$-NMR spectrum ($CDCl_3$, 100 MHz, 298 K).

a)

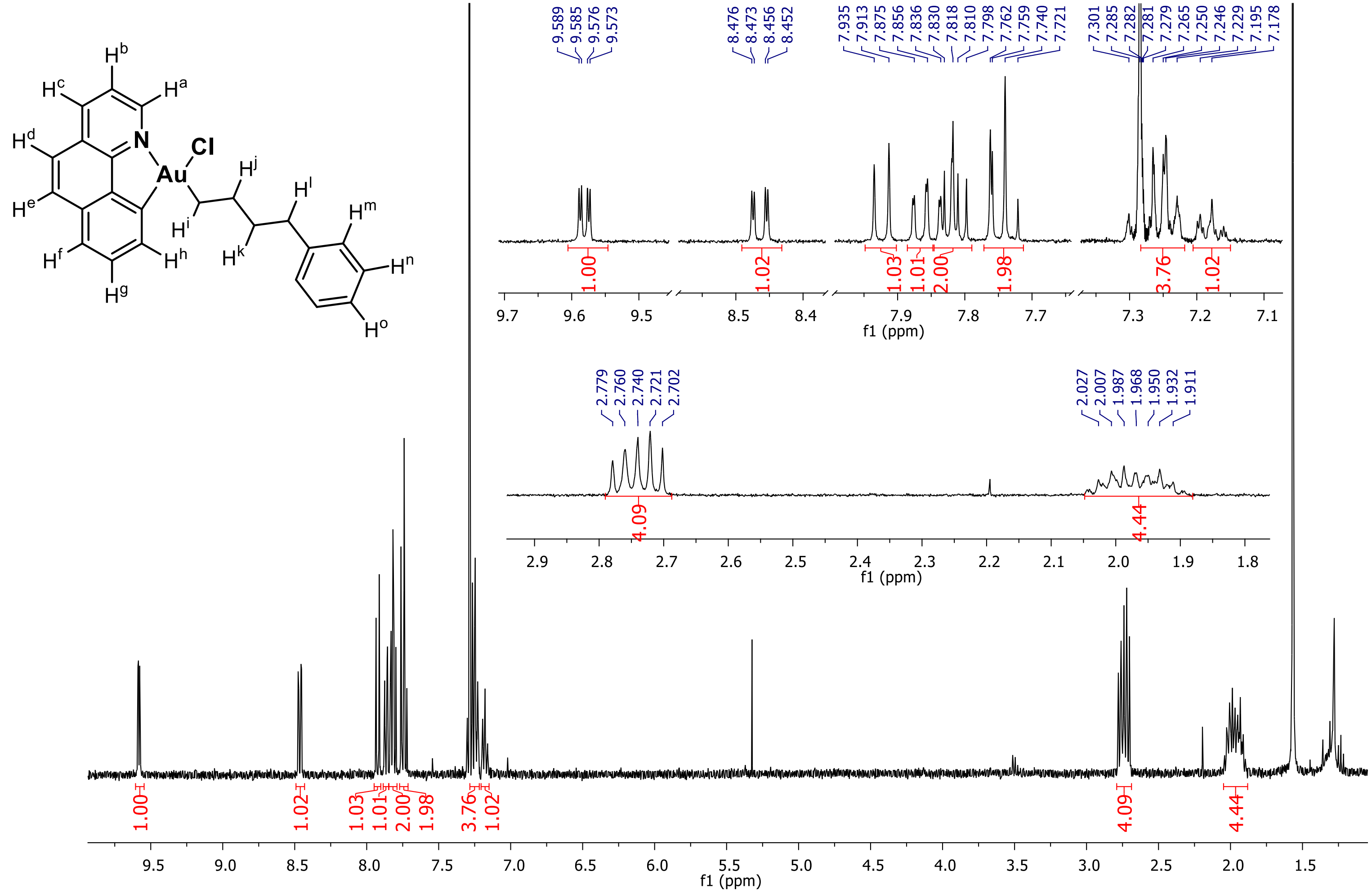

b)

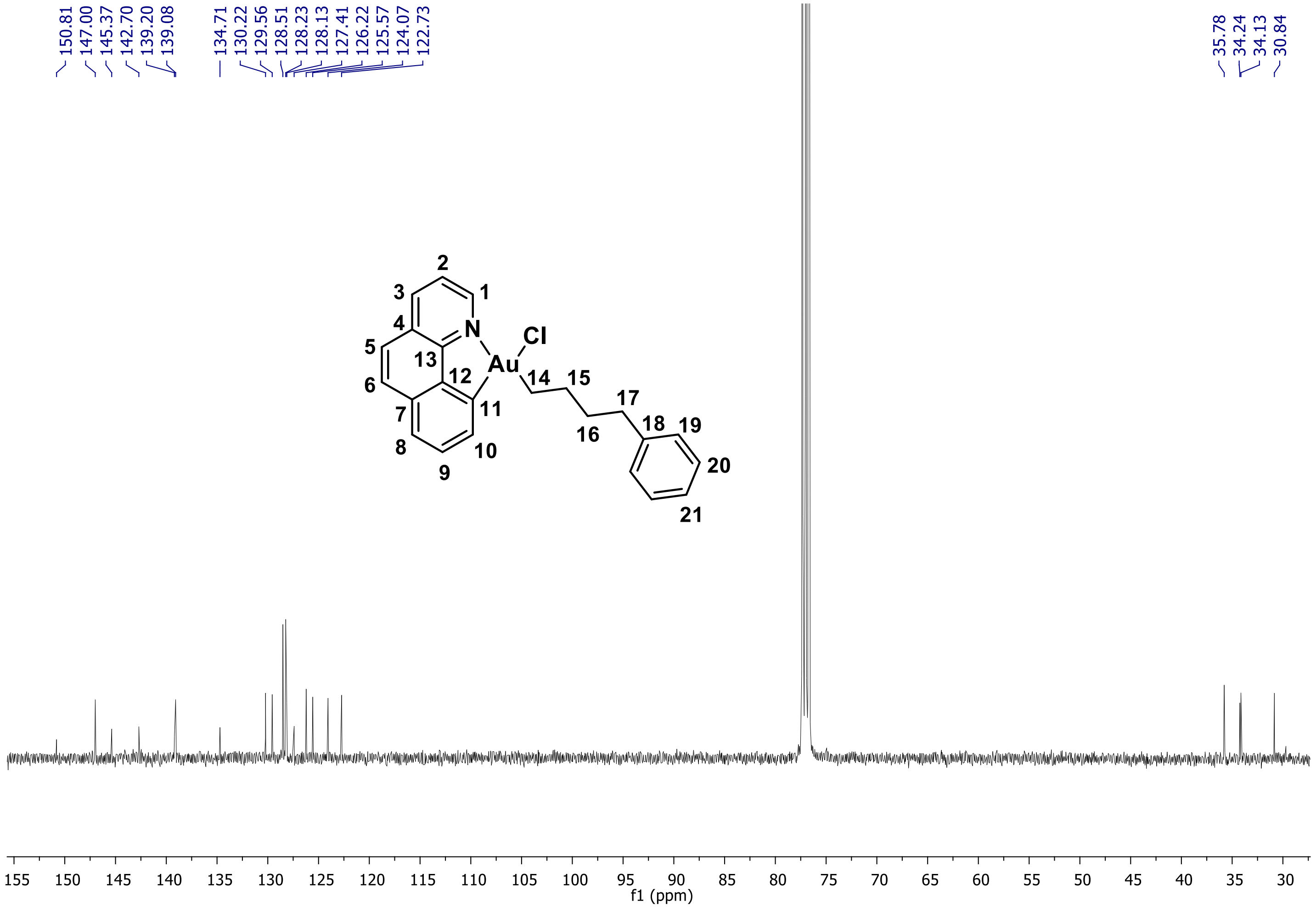
150.81
147.00
145.37
142.70
139.20
139.08
134.71
130.22
129.56
128.51
128.23
128.13
127.41
126.22
125.57
124.07
122.73
35.78
34.24
34.13
30.84
N
Cl
Au
1
2
3
4
5
6
7
8
9
10
11
12
13
14
15
16
17
18
19
20
21
155 150 145 140 135 130 125 120 115 110 105 100 95 90 85 80 75 70 65 60 55 50 45 40 35 30
f1 (ppm)

Figure S4. a) ^{1}H-NMR spectrum of complex **5A-Cl-*d₄*** in CD_2Cl_2, 500 MHz, at 298 K (crude); b) ^{1}H-NMR spectrum of complex **5A-Cl-*d₄*** in CD_2Cl_2, 300 MHz, at 298 K (purified) ; c) $^{13}C\{^1H\}$-NMR spectrum in CD_2Cl_2, 125 MHz at 298 K (crude).

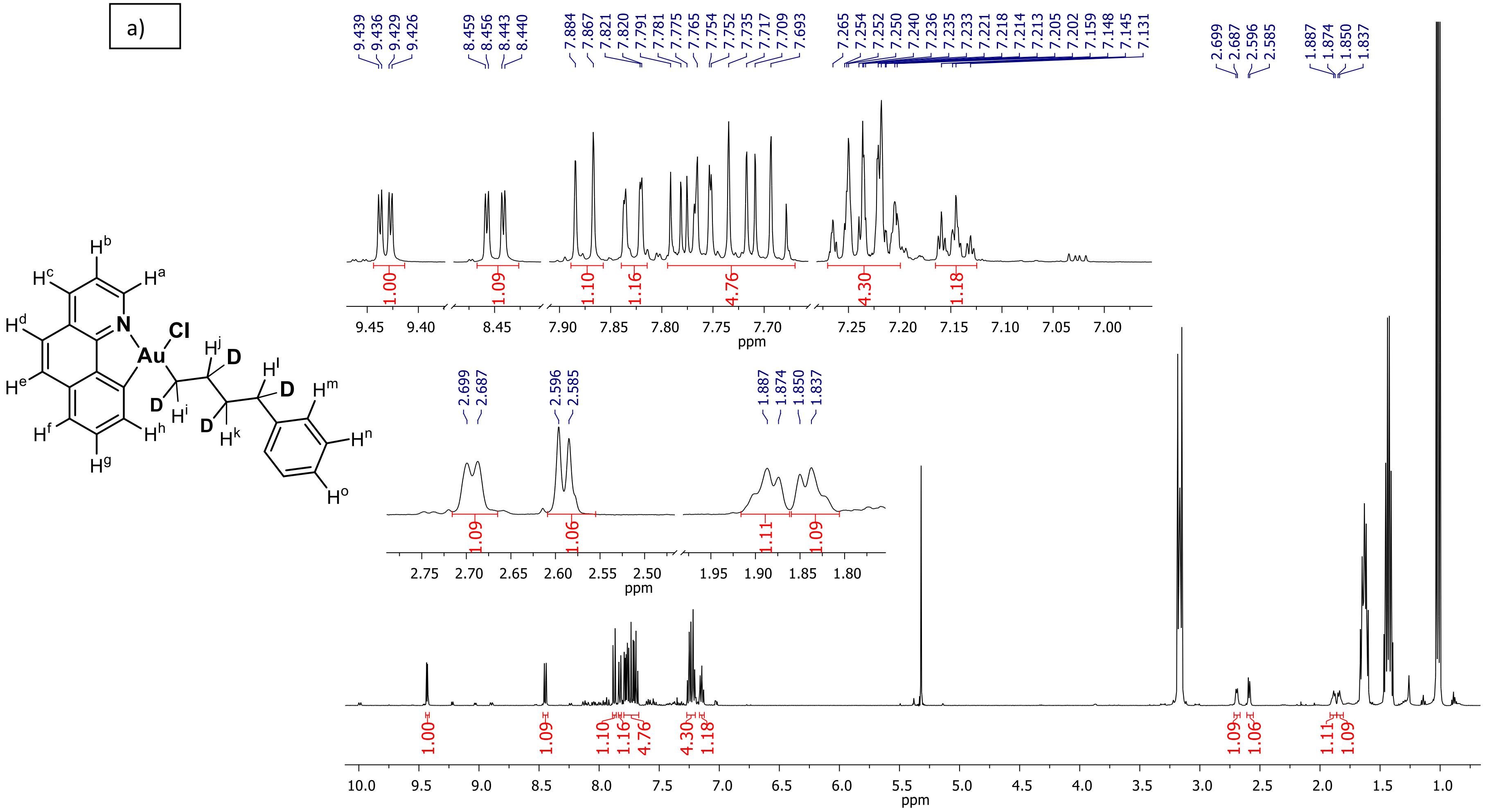

b)

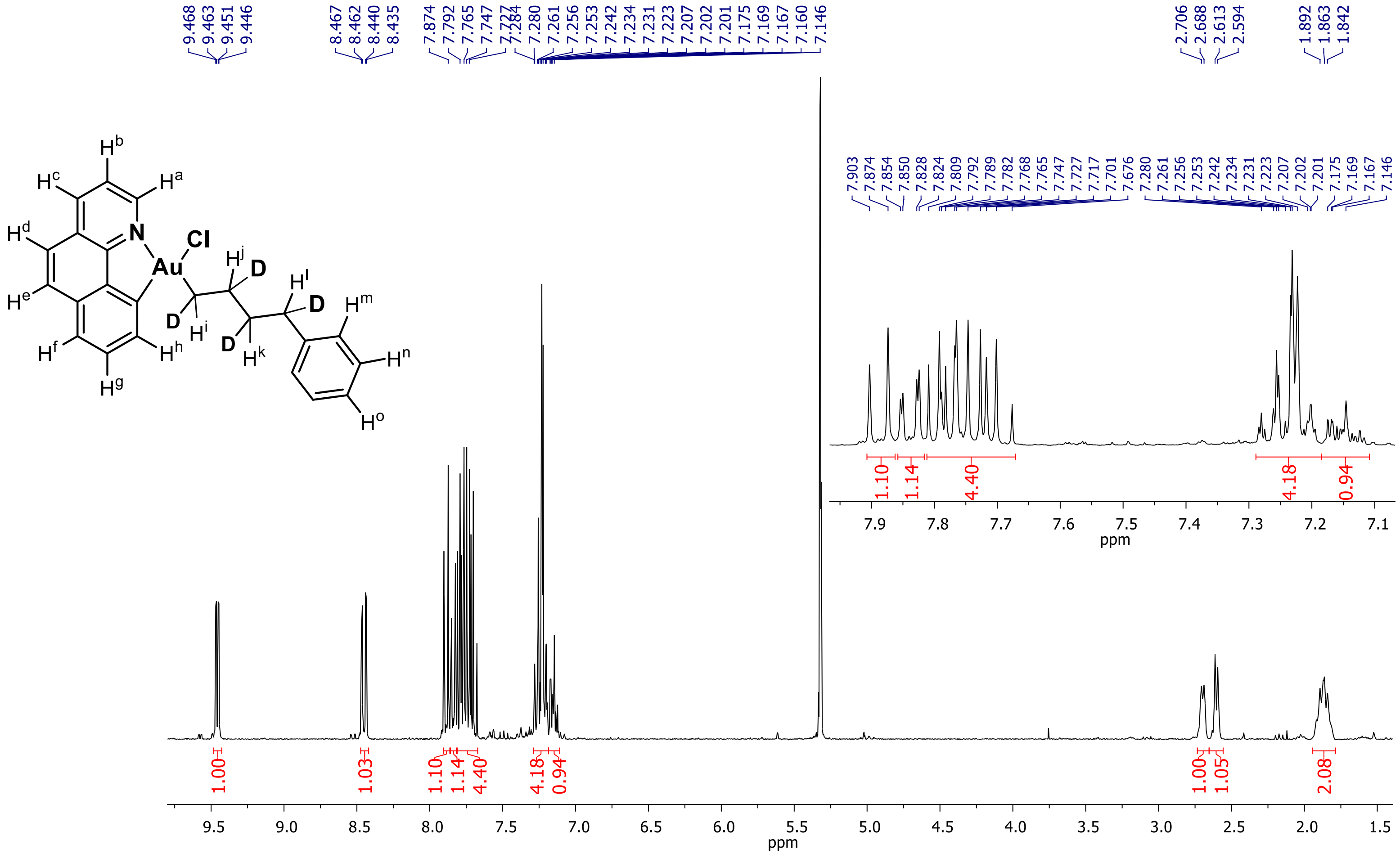

9.468
9.463
9.451
9.446
8.467
8.462
8.440
8.435
7.874
7.792
7.765
7.747
7.727
7.284
7.280
7.261
7.256
7.253
7.242
7.234
7.231
7.223
7.207
7.202
7.201
7.175
7.169
7.167
7.160
7.146
2.706
2.688
2.613
2.594
1.892
1.863
1.842
7.903
7.874
7.854
7.850
7.828
7.824
7.809
7.792
7.789
7.782
7.768
7.765
7.747
7.727
7.717
7.701
7.676
7.280
7.261
7.256
7.253
7.242
7.234
7.231
7.223
7.207
7.202
7.201
7.175
7.169
7.167
7.146
1.10
1.14
4.40
4.18
0.94
ppm
1.00
1.03
1.10
1.14
4.40
4.18
0.94
1.00
1.05
2.08
ppm
Au
Cl
N
D

c)

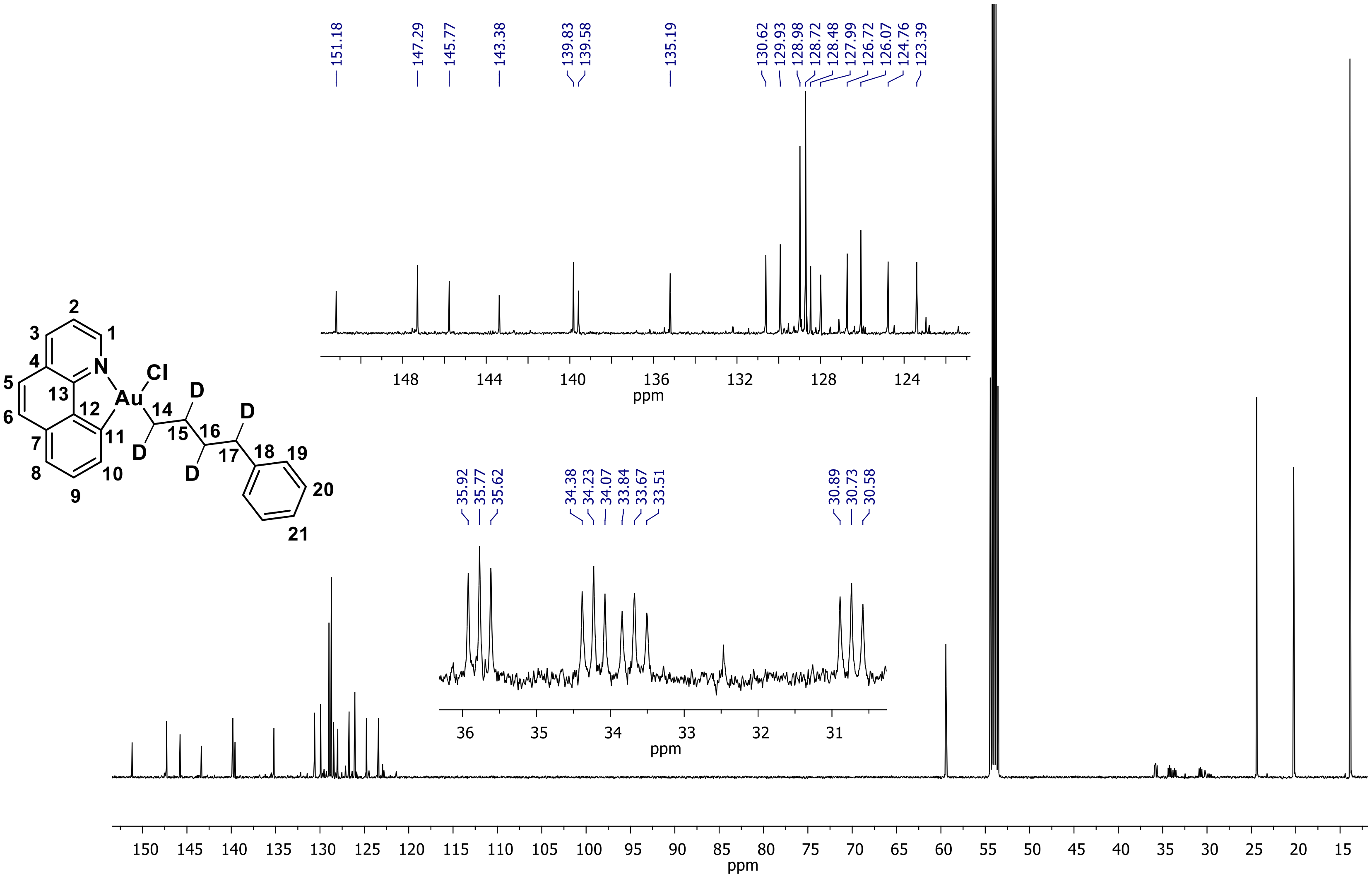
151.18
147.29
145.77
143.38
139.83
139.58
135.19
130.62
129.93
128.98
128.72
128.48
127.99
126.72
126.07
124.76
123.39
148
144
140
136
132
128
124
ppm
35.92
35.77
35.62
34.38
34.23
34.07
33.84
33.67
33.51
30.89
30.73
30.58
36
35
34
33
32
31
ppm
150
145
140
135
130
125
120
115
110
105
100
95
90
85
80
75
70
65
60
55
50
45
40
35
30
25
20
15
ppm
Au
Cl
N
D
1
2
3
4
5
6
7
8
9
10
11
12
13
14
15
16
17
18
19
20
21

Figure S5. a) ^{1}H-NMR spectrum of complex **6A** in CD_2Cl_2, 400 MHz, at 298 K; b) $^{13}C\{^{1}H\}$-NMR spectrum (CD_2Cl_2, 100 MHz, 298 K).

a)

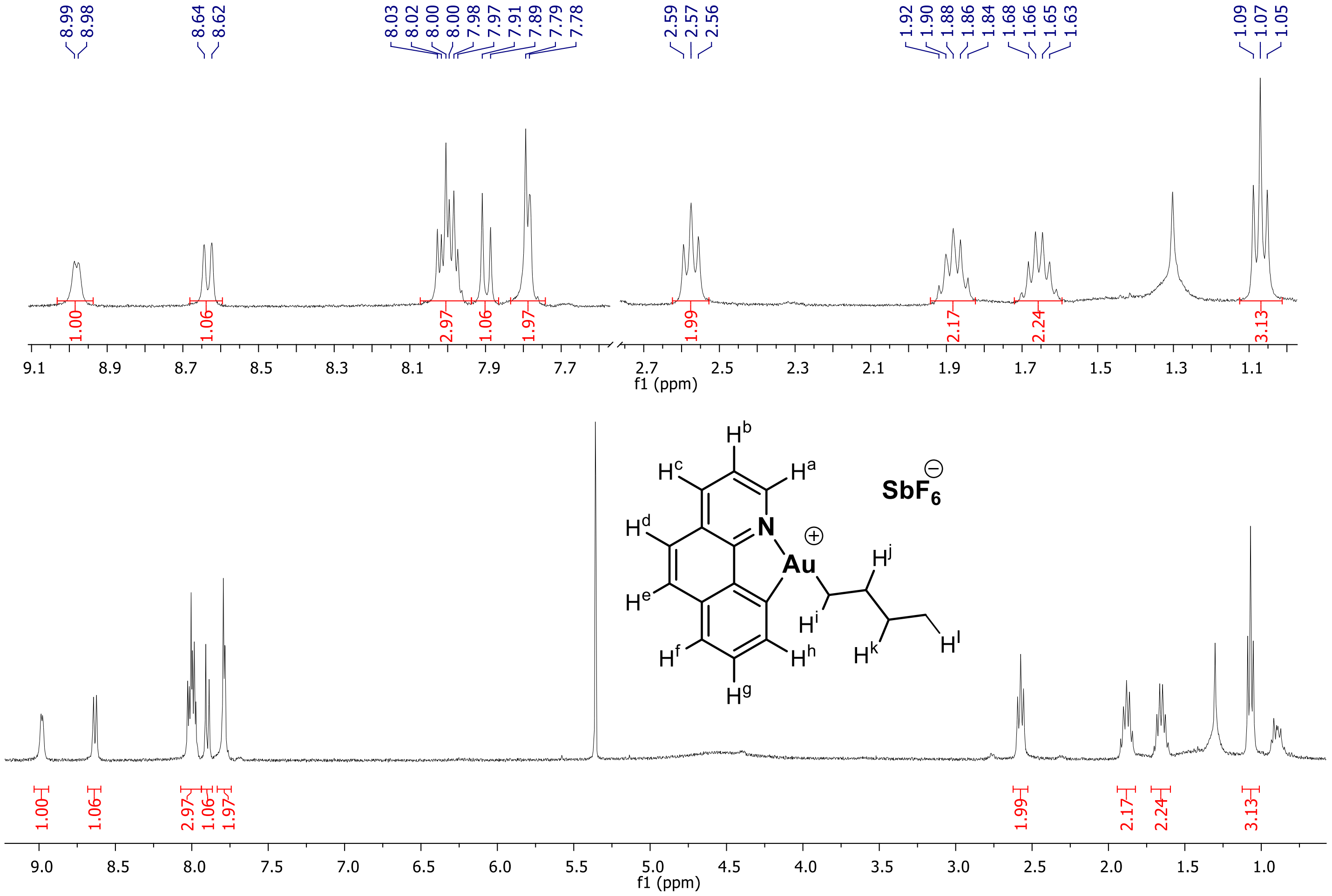

b)

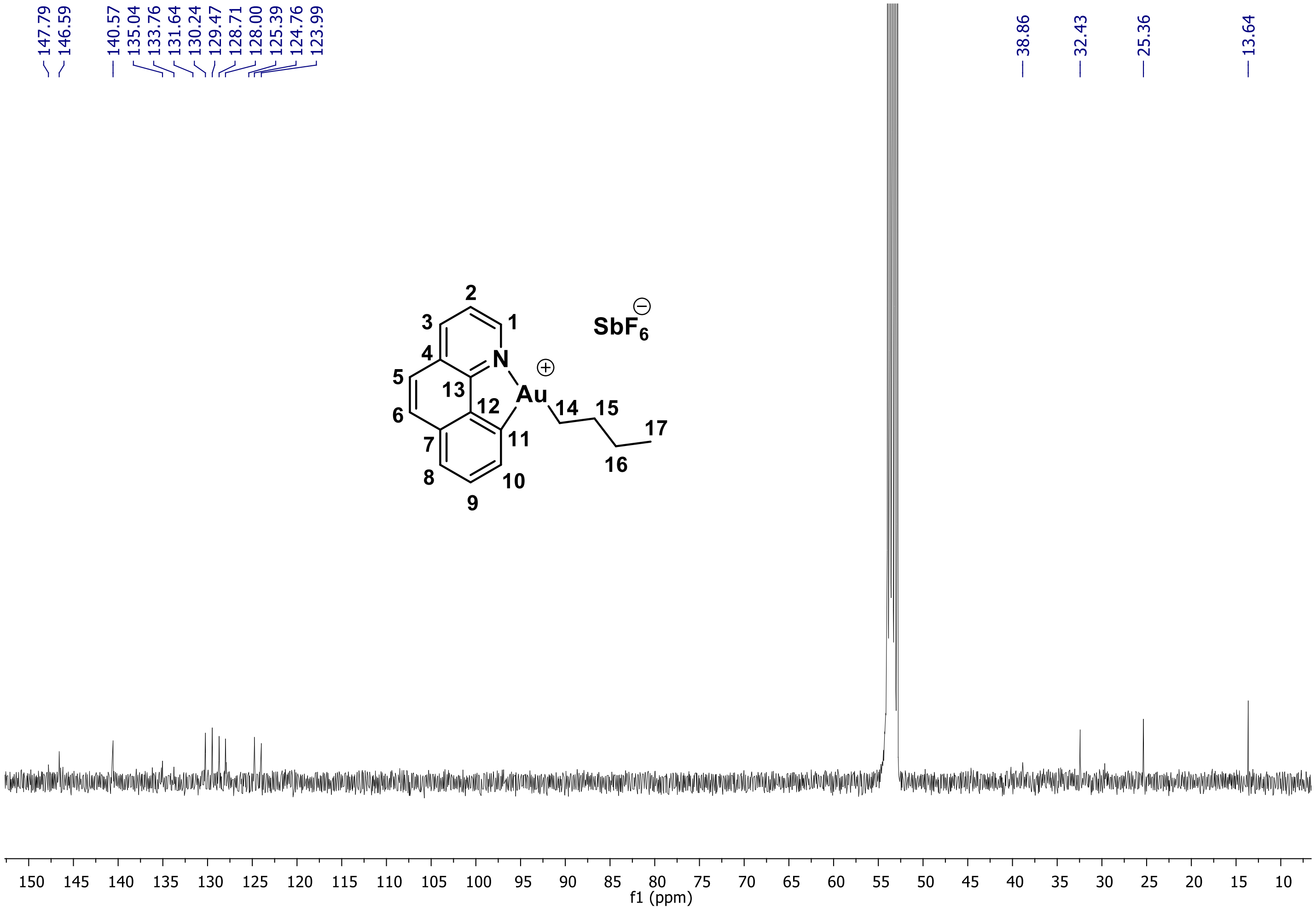
147.79
146.59
140.57
135.04
133.76
131.64
130.24
129.47
128.71
128.00
125.39
124.76
123.99
38.86
32.43
25.36
13.64
SbF6
Au
N
1
2
3
4
5
6
7
8
9
10
11
12
13
14
15
16
17
150 145 140 135 130 125 120 115 110 105 100 95 90 85 80 75 70 65 60 55 50 45 40 35 30 25 20 15 10
f1 (ppm)

Figure S6. a) ^{1}H-NMR spectrum of complex **6-Cl** in $CDCl_3$, 400 MHz, at 298 K; b) $^{13}C\{^1H\}$-NMR spectrum ($CDCl_3$, 100 MHz, 298 K).

a)

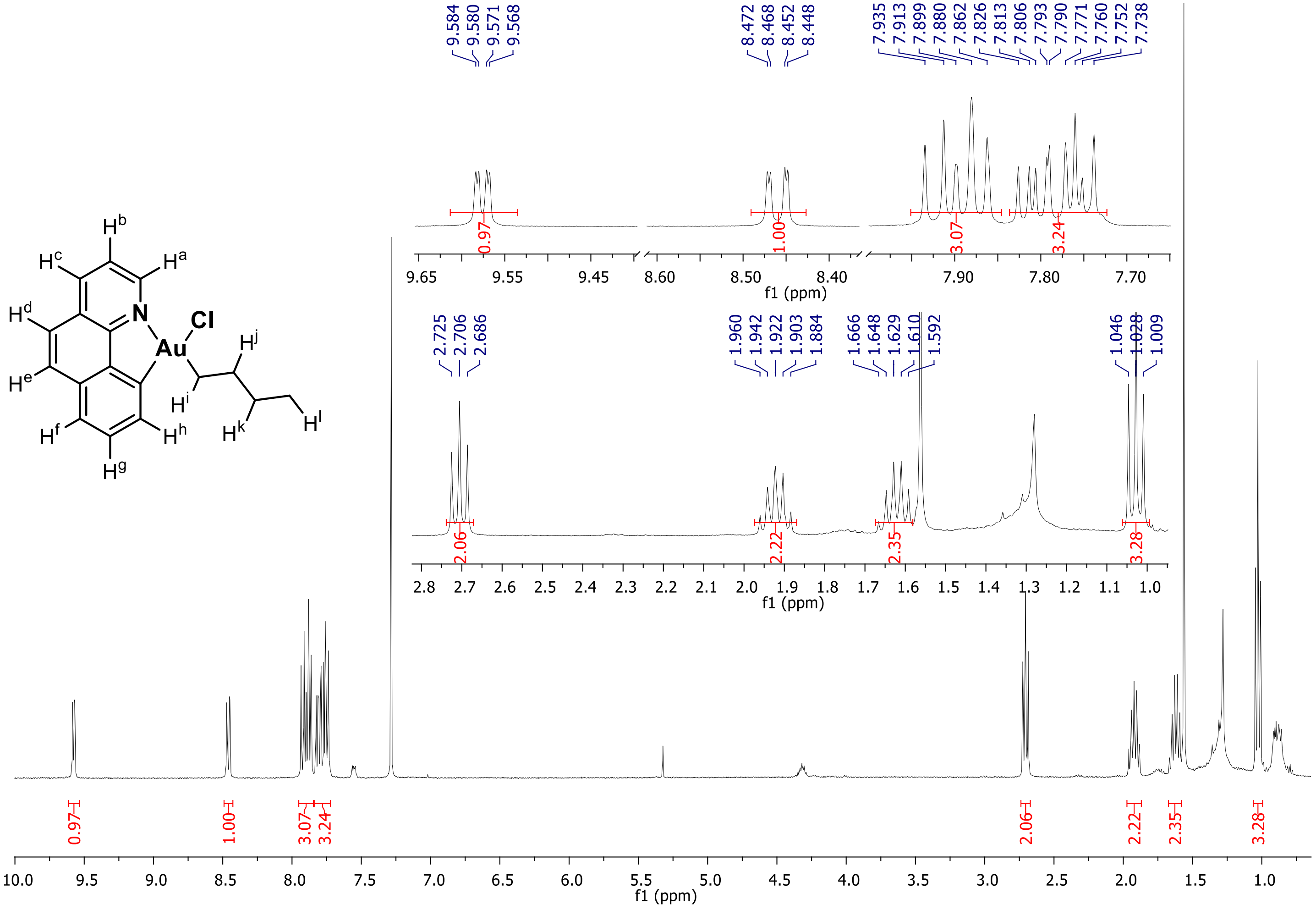

b)

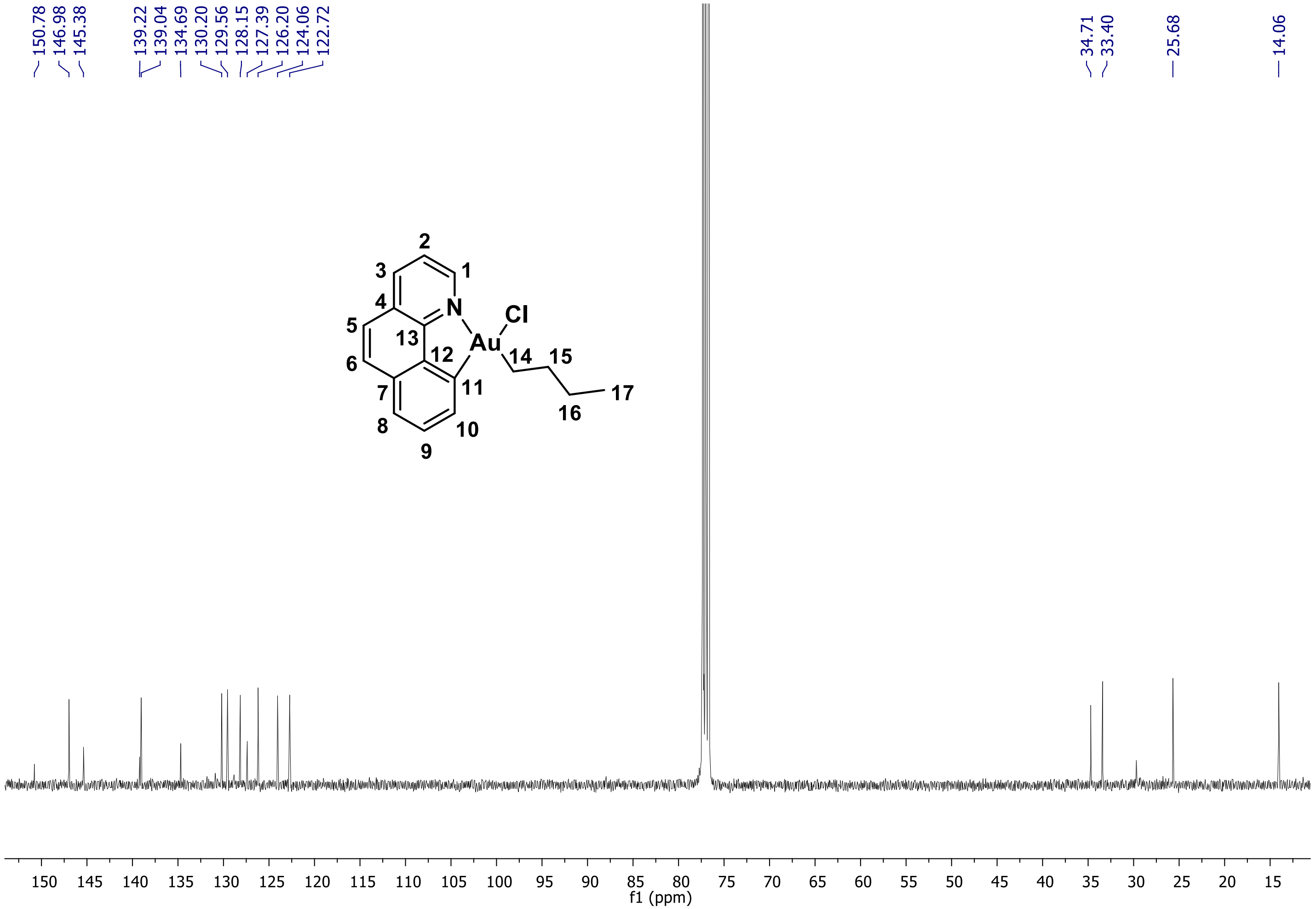
150.78
146.98
145.38
139.22
139.04
134.69
130.20
129.56
128.15
127.39
126.20
124.06
122.72
34.71
33.40
25.68
14.06
N
Au
Cl
1
2
3
4
5
6
7
8
9
10
11
12
13
14
15
16
17
150 145 140 135 130 125 120 115 110 105 100 95 90 85 80 75 70 65 60 55 50 45 40 35 30 25 20 15
f1 (ppm)

Figure S7. Full energy profile (ΔG and ΔE values into brackets, in kcal/mol) computed at the PCM(Dichloromethane)-B3PW91/SDD+f(Au)/6-31G**(other atoms) level of theory for different insertions of ethylene and β–hydride elimination from the [(N,C)AuPh]$^+$ complex **1A** (*cis* and *trans* isomers).

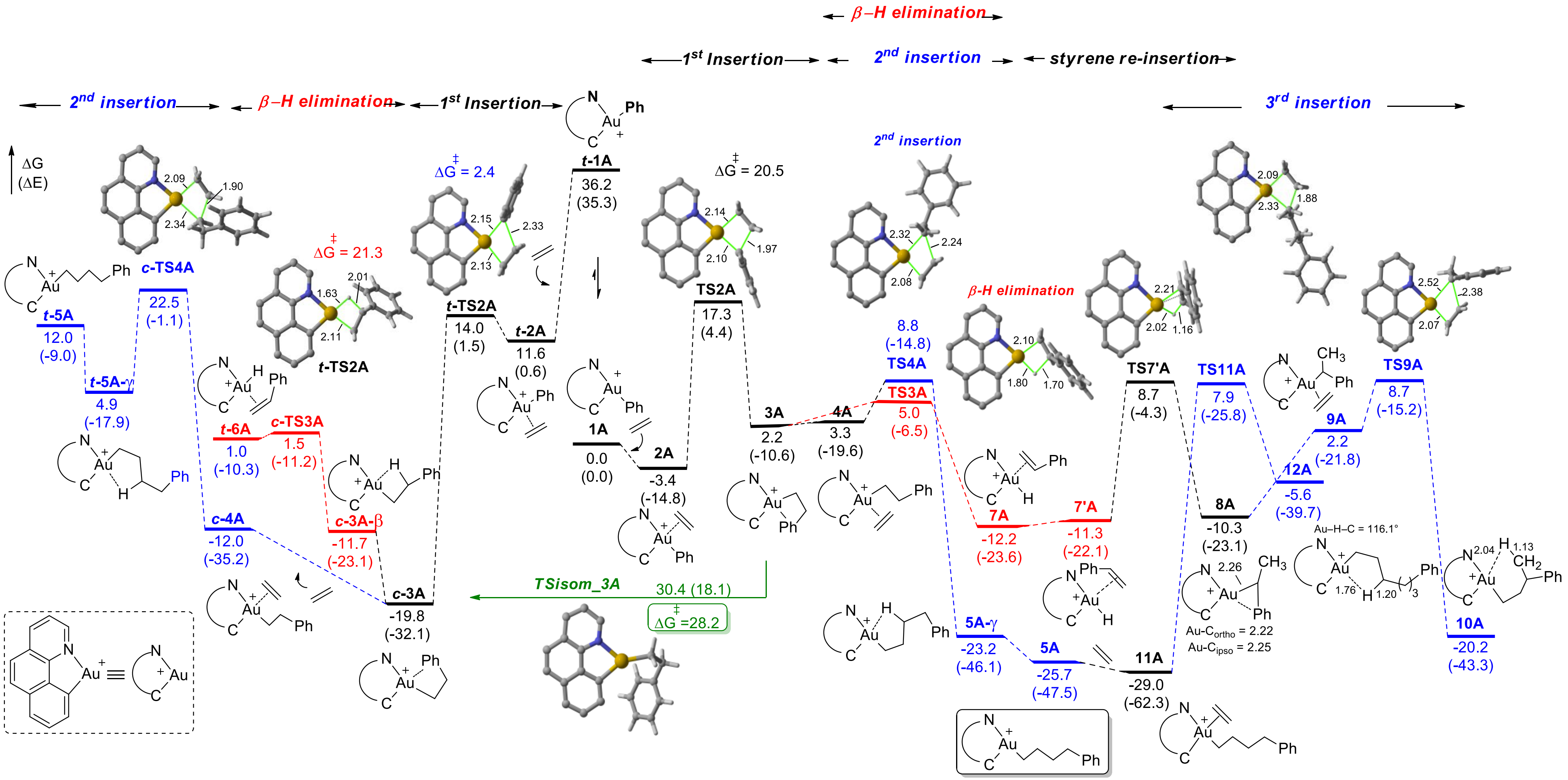

Figure S8. Key structures and selected geometric parameters (distances in Å) for the insertion of ethylene into the Au-Ph bond of complex **1A** at the PCM(Dichloromethane)-B3PW91/SDD+f(Au)/6-31G**(other atoms) level of theory.

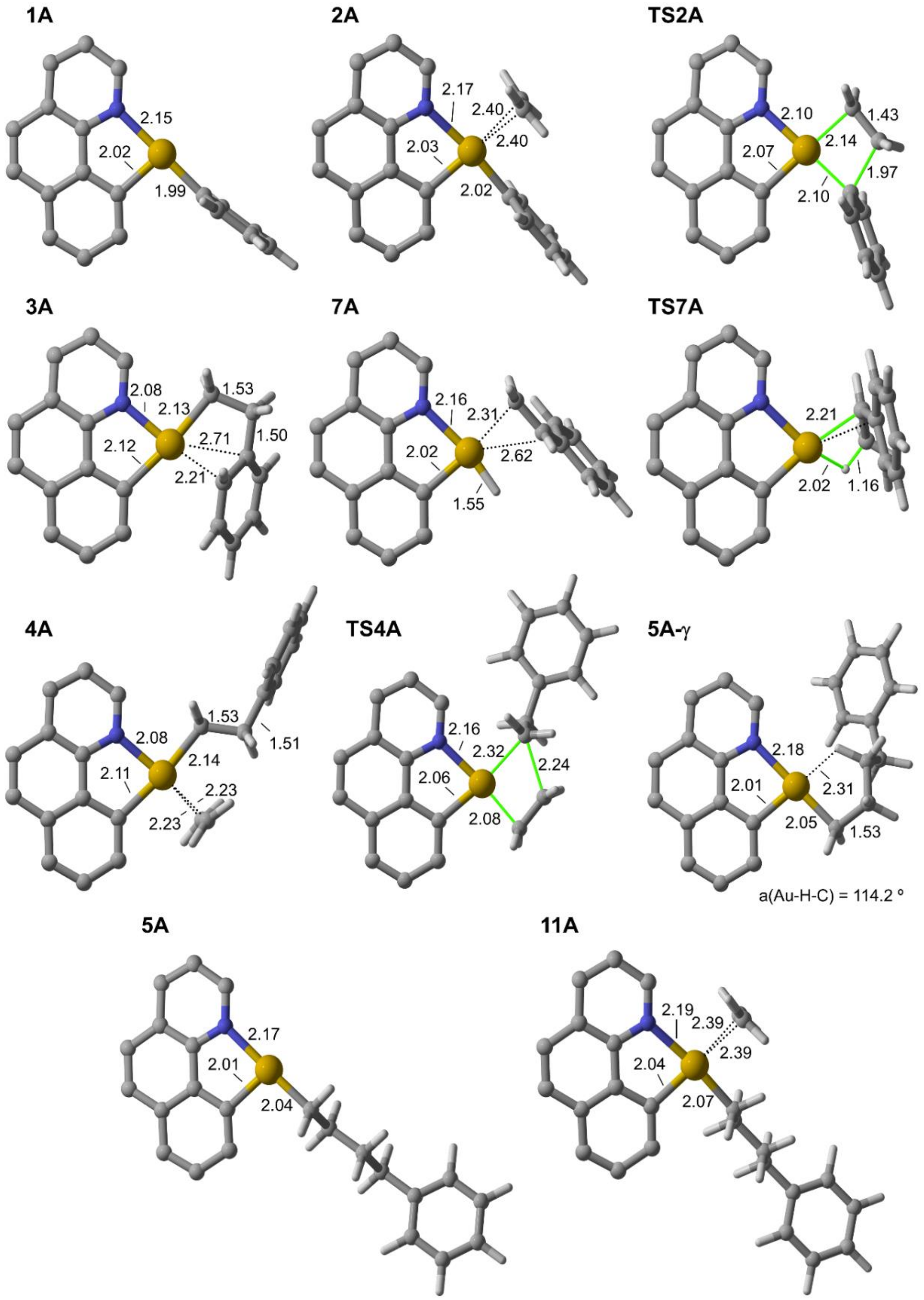

Table S1. NBO analysis of the π-ethylene coordination in complex **2A**, of the π-arene coordination in complex **3A** and of the γ-CH agostic interaction in complex **5A-γ** at the PCM(Dichloromethane)-B3PW91/SDD+f(Au)/6-31G**(other atoms) level of theory. Relative stability of complexes **2A** and **2A_parallel** in kcal/mol.

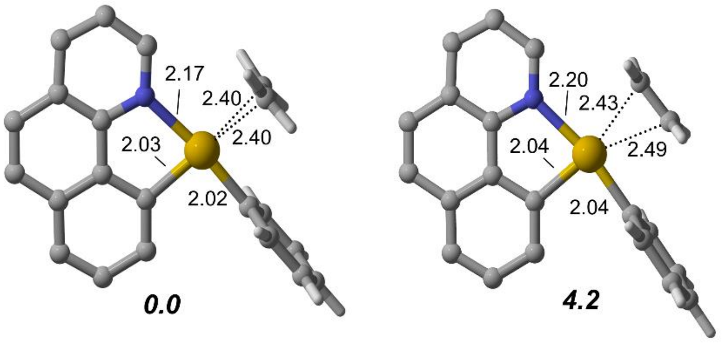

2A   2A_parallel

Complex	Donation[a]	Back-Donation[a]	NLMO[b]
**2A**<br>*ethylene perpendicular*	45.7<br>($\pi_{CC}\rightarrow\sigma^*_{AuC}$)	14.1<br>($d_{Au}\rightarrow \pi^*_{CC}$)	43.7 % C<br>43.7 % C<br>6.5% Au<br>5.6% C_{napht}
**2A_parallel**<br>*ethylene parallel*	50.9<br>($\pi_{CC}\rightarrow\sigma^*_{AuC}$)	9.7<br>($d_{Au}\rightarrow \pi^*_{CC}$)	40.2 % C<br>48.1% C<br>6.0 % Au<br>5.0 % % C_{napht}
**3A**	31.0<br>($\pi_{CC}\rightarrow\sigma^*_{AuN}$)	--<br>($d_{Au}\rightarrow \pi^*_{CC}$)	22.8 % C_{ipso}<br>50.5 % C_{ortho}<br>1.5% N<br>13.0% Au
**5A-γ**	7.1<br>($\sigma_{CH}\rightarrow\sigma^*_{AuC}$)	2.3<br>($\sigma_{AuC}\rightarrow \sigma^*_{C-H}$)	57.0 % C<br>40.2 % H<br>1.1 % Au

[a] Stabilizing energy ΔE(2) in kcal/mol. [b] Participation of each atom (%) in the NLMO $\pi_{C=C}$ and the NLMO σ_{CH}.

Figure S9. Energy profiles (ΔG and ΔE values into brackets, in kcal/mol) computed at the PCM(Dichloromethane)-B3PW91/SDD+f(Au)/6-31G**(other atoms) level of theory for the third ethylene insertion from **5A** and second ethylene insertion from **8A**(see complete profile in Figure S13).

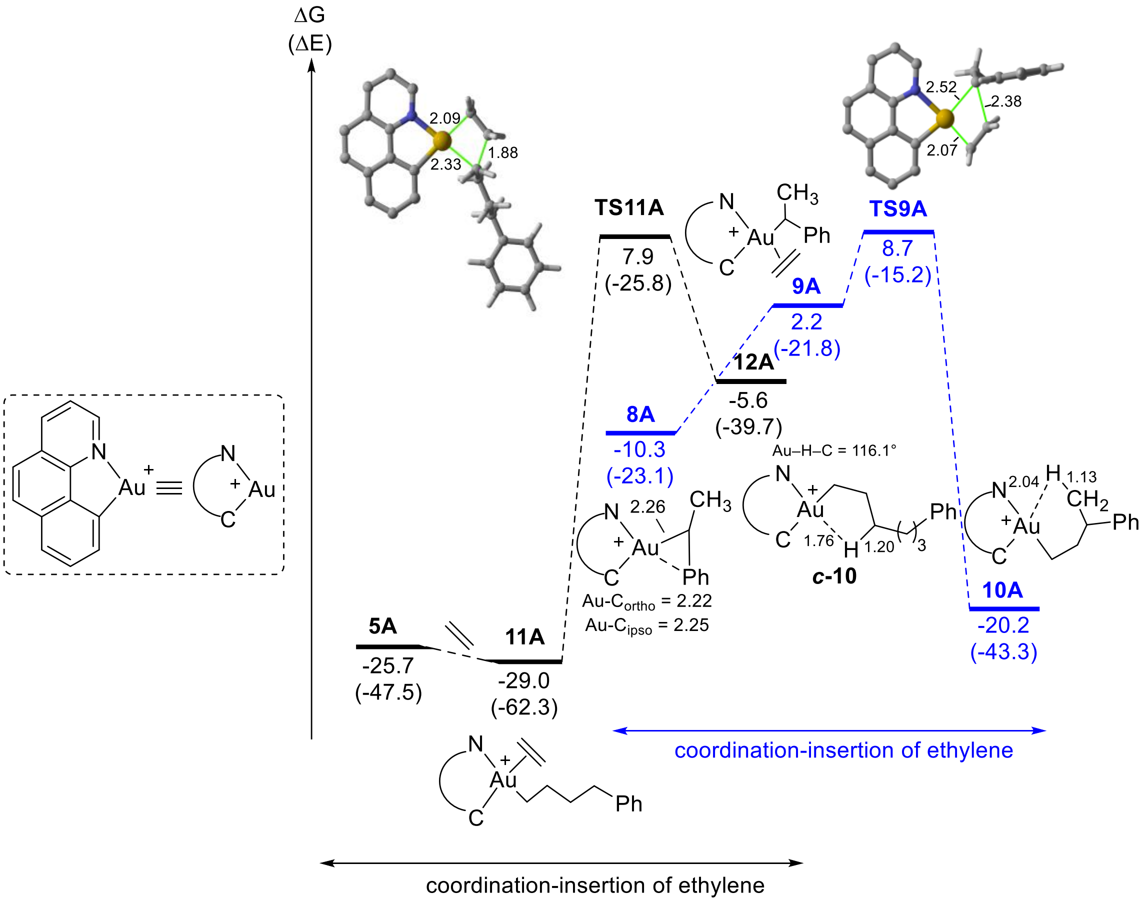

Figure S10. Energy profiles (ΔG and ΔE values into brackets, in kcal/mol) computed at the PCM(Dichloromethane)-B3PW91/SDD+f(Au)/6-31G**(other atoms) level of theory for β- andγ-hydride eliminations from complex **5A**.

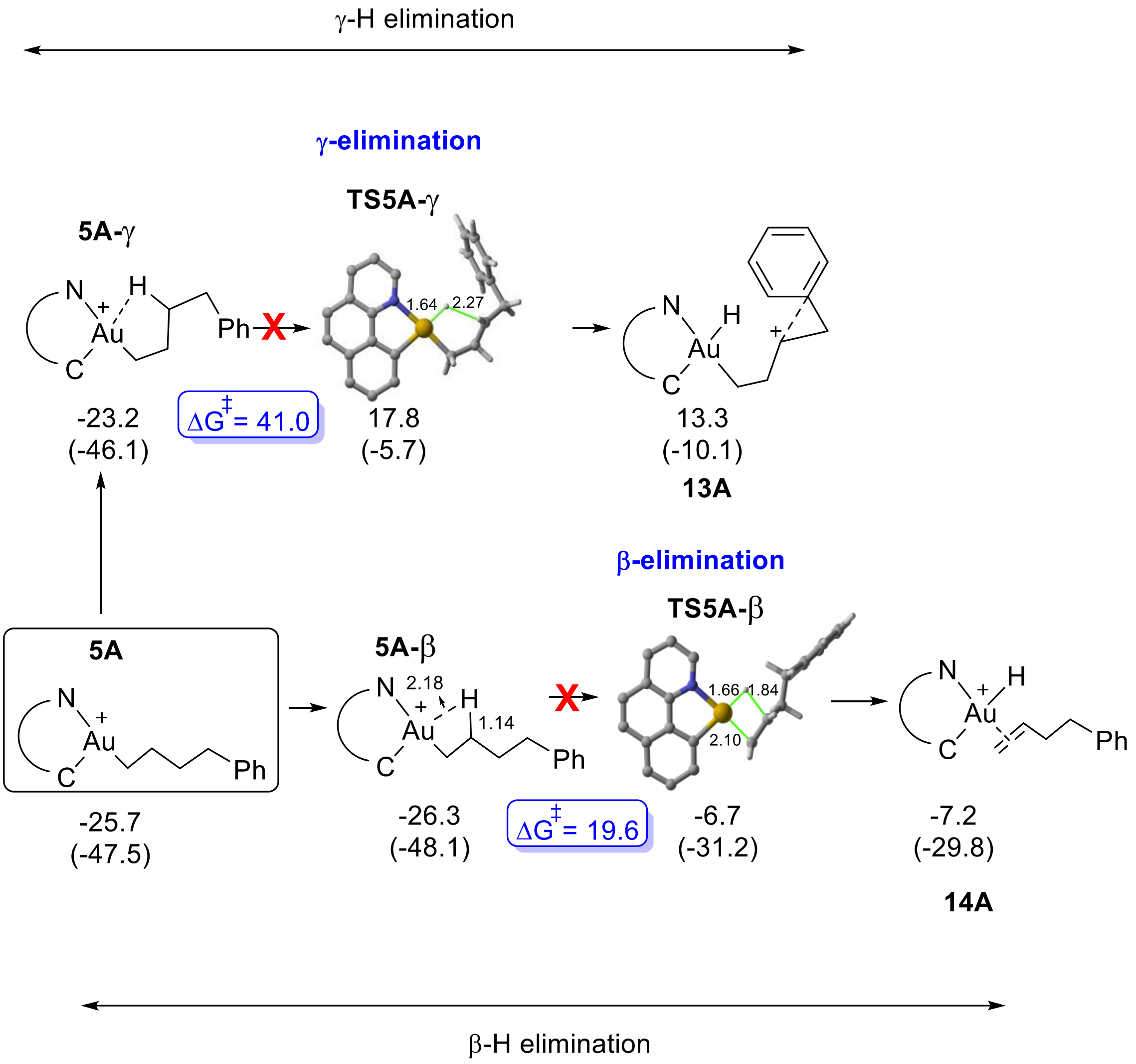

Figure S11. Energy profile (ΔG and ΔE values in kcal/mol) computed at the PCM(Dichloromethane)-B3PW91/SDD+f(Au)/6-31G**(other atoms) level of theory for β-hydride and 2nd insertion from ***t*-3A** (see complete profile in Figure S13).

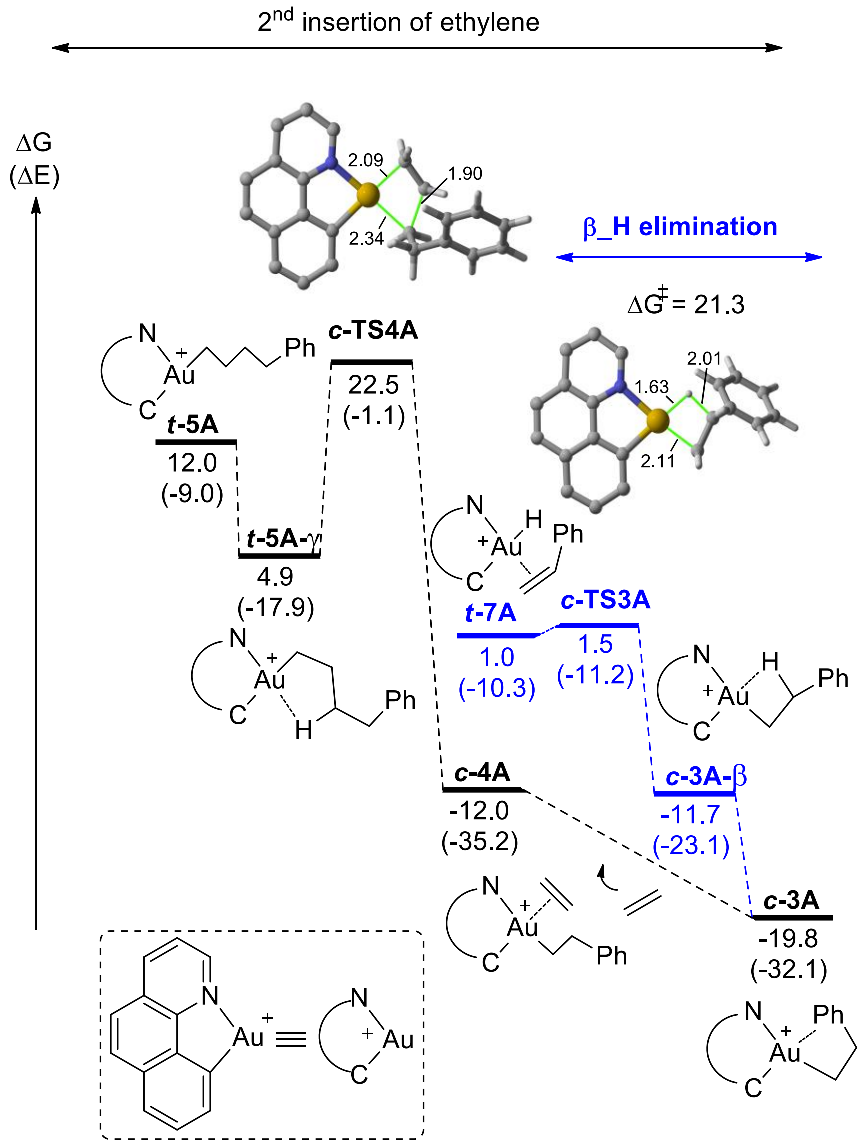

Figure S12. Energy profile (ΔG and ΔE values in kcal/mol) computed at the PCM(Dichloromethane)-B3PW91/SDD+f(Au)/6-31G**(other atoms) level of theory for ethylene insertion and β-hydride elimination from the $[(P,C)AuPh]^+$ complex **1C**.

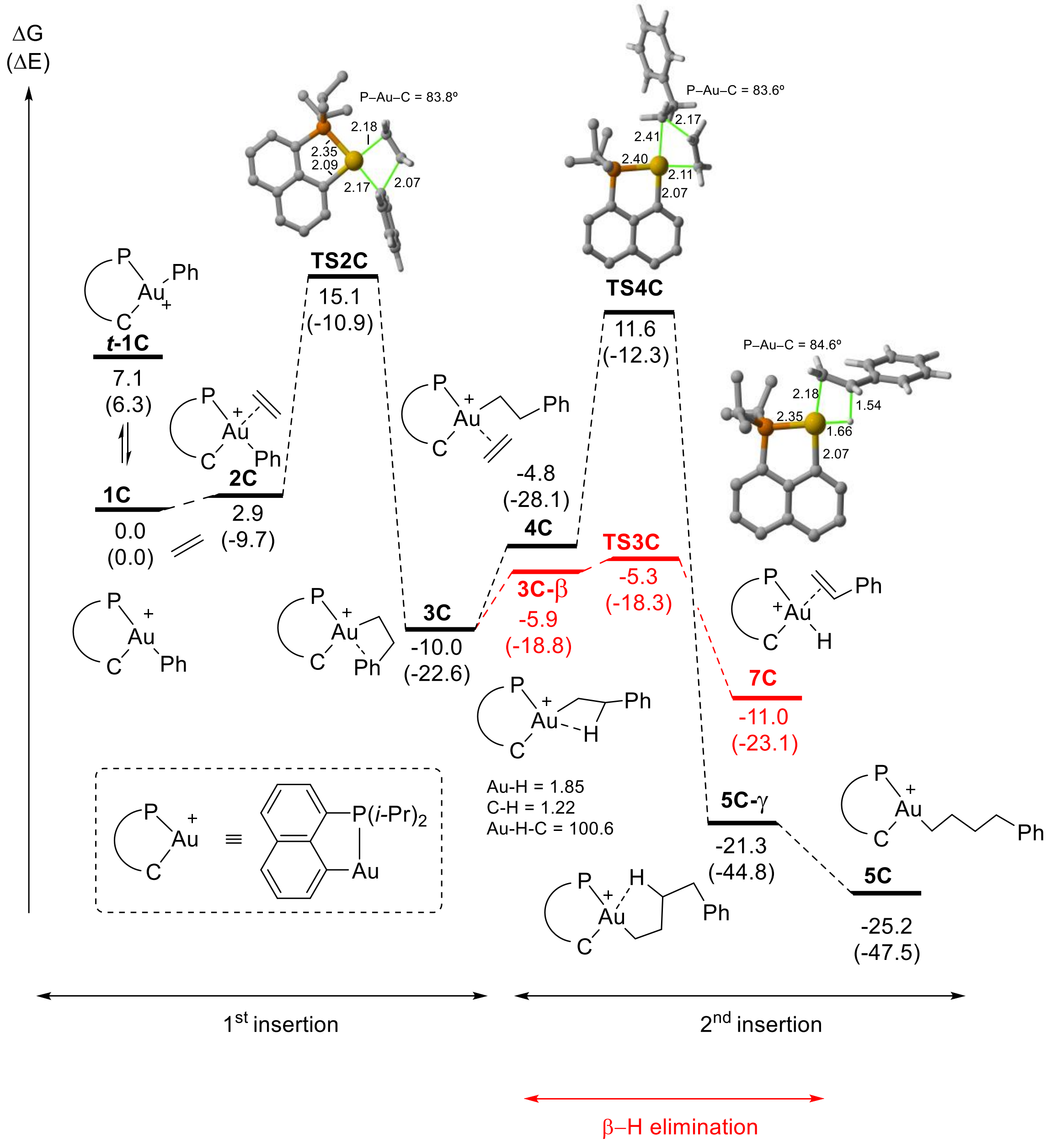

Figure S13. Key structures and selected geometric parameters (distances in Å) for the two first insertions of ethylene into the Au-Ph bond of complex **1C** and β-hydride elimination from **3C** at the PCM(Dichloromethane)-B3PW91/SDD+f(Au)/6-31G**(other atoms) level of theory.

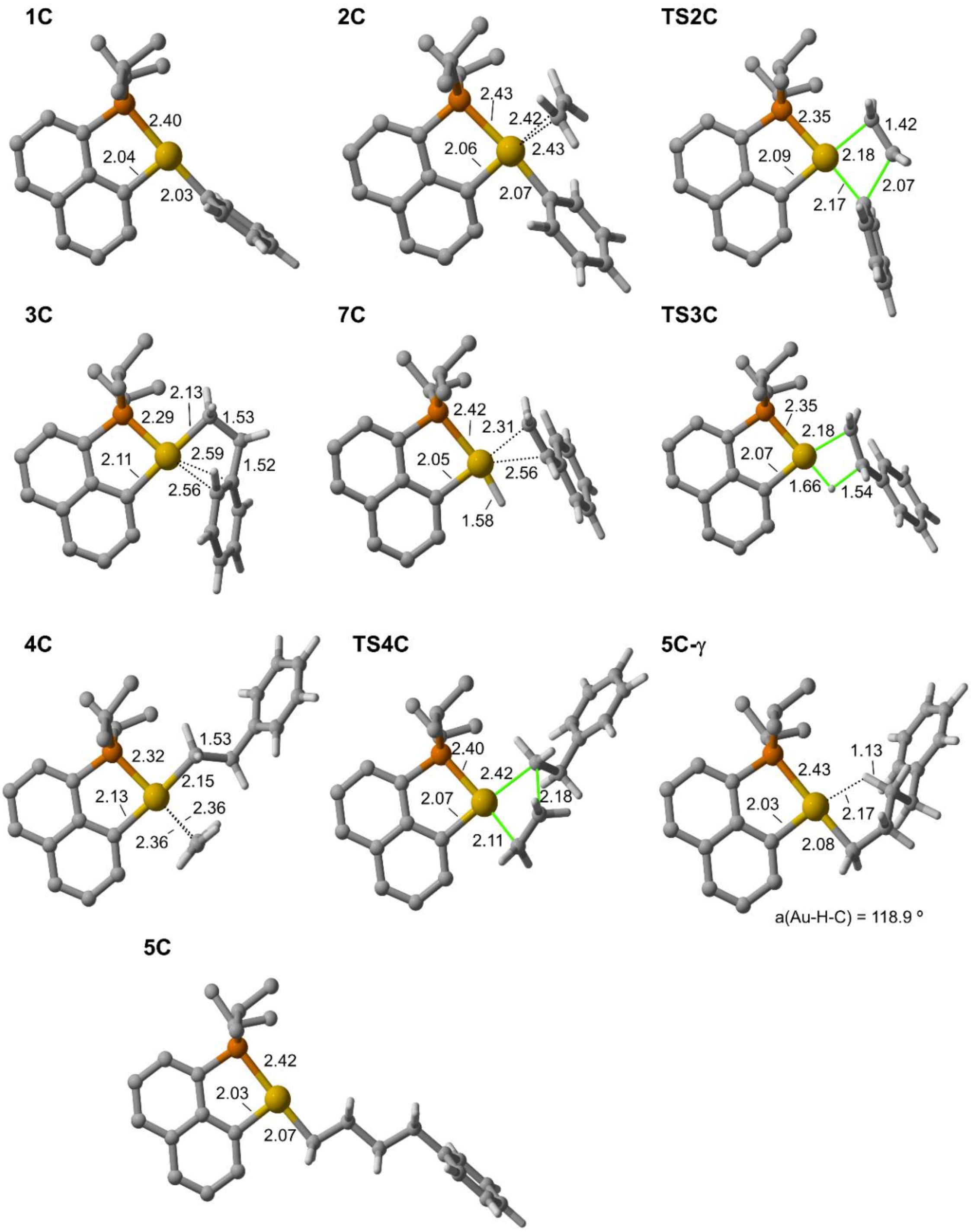

Figure S14. Reaction paths (ΔG and ΔE values, into brackets in kcal/mol) computed at the PCM(Dichloromethane)-B3PW91/SDD+f(Au)/6-31G**(other atoms) level of theory for the reductive elimination (C_{sp2}-H coupling) of the gold(III) hydride complexes **7A** and **7C**.

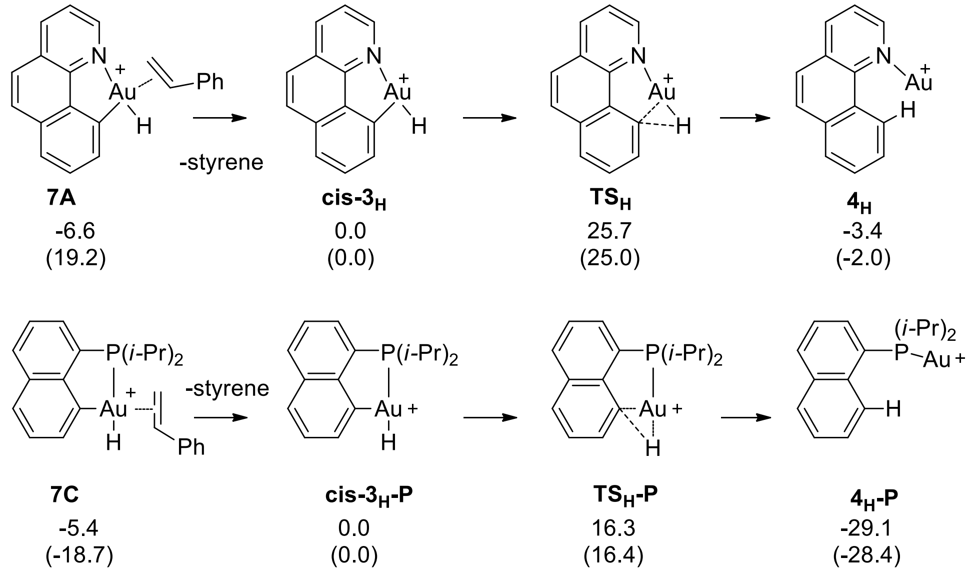

Table S2. NBO analysis of the π-ethylene coordination in complex **2C**, of the π-arene coordination in complex **3C** and of the γ-CH agostic interaction in complex **5C-γ** at the PCM(Dichloromethane)-B3PW91/SDD+f(Au)/6-31G**(other atoms) level of theory. Relative stability of complexes **2C** and **2C_parallel** in kcal/mol.

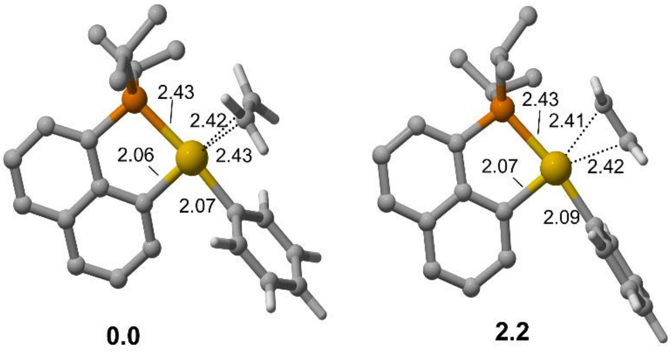

2C **2C_parallel**

Complex	Donation	Back-Donation	NLMO
**2C**<br>*ethylene perpendicular*	34.3<br>($\pi_{CC}\rightarrow\sigma^*_{AuC}$)	13.0<br>($d_{Au}\rightarrow \pi^*_{CC}$)	42.4 % C<br>44.2 % C<br>6.6 % Au<br>5.9 % C_{napht}
**2C_parallel**<br>*ethylene parallel*	34.3<br>($\pi_{CC}\rightarrow\sigma^*_{AuC}$)<br>**parallel**	12.5<br>($d_{Au}\rightarrow \pi^*_{CC}$)	40.2 % C<br>45.7 % C<br>6.9 % Au<br>6.2 % C_{napht}
**3C**	6.4<br>($\pi_{CC}\rightarrow\sigma^*_{AuP}$)	4.0<br>($d_{Au}\rightarrow \pi^*_{CC}$)	37.2 % C_{ipso}<br>42.4 % C_{ortho}<br>4.5 % Au<br>3.4 % P
**5C-γ**	2.2	12.0<br>($\sigma_{C-H}\rightarrow\sigma^*_{AuC}$)	55.6 % C<br>40.5 % H<br>1.5 % Au

4. X-ray Crystallography Data

Crystallographic data for complexes**5A-H_2O** (CCDC-1574199) and**5A-Cl** (CCDC-1574200) can be obtained free of charge from the Cambridge Crystallographic Data Centre (CCDC) *via*www.ccdc.cam.ac.uk/data_request/cif.

Colorless crystals were grown from a CD_2Cl_2 solution of the compound for **5A-H_2O**, and from slow diffusion of pentane in a $CHCl_3$ solution of the **5A-Cl** complex. The data were collected at low temperature (193(2)K) on a Bruker-AXS APEX II QUAZAR diffractometer equipped with a 30W air-cooled microfocus source for **5A-H_2O**and on a Bruker-AXS PHOTON100 D8 VENTURE diffractometer for **5A-Cl**,using MoKα radiation (λ= 0.71073Å). Phi- and omega-scans were used. An empirical absorption correction with SADABS was applied.[17] The structures were solved by direct methods (SHELXS-97)[18] or using Intrinsic phasing method (SHELXT)[19] and refined by full-matrix least squares on F^2.[17]All non-H atoms were refined with anisotropic displacement parameters.

17 SADABS. Bruker AXS Inc., Madison, Wisconsin, USA.
18 Sheldrick, G. M., *Acta Cryst*. **2008**, A64, 112-122.
19 Sheldrick, G. M., *Acta Cryst*. **2015**, A71, 3-8.

Table S3. Key structural parameters of complex **5A-H$_2$O**.

**Chemical formula**	$C_{23}H_{23}Au_1N_1O_1^{1+},F_6Sb_1^{1-}$
**fw (g mol^{-1})**	762.15
**Bond precision**	C-C = 0.0083 Å
**T (K)**	193
***a* (Å)**	6.6287(3)
***b* (Å)**	16.4246(8)
***c* (Å)**	21.5858(11)
**α (deg.)**	90
**β (deg.)**	90
**γ (deg.)**	90
***V* (Å^3)**	2350.1(2)
**$\rho_{calcd.}$ (g cm^{-3})**	2.154
**λ (Å)**	0.71073
**Mu (mm-1)**	7.448
**F000**	1440.0
**F000’**	1430.69
**h,k,lmax**	9,22,29
**Nref**	6595[3740]
**R$_1$ [I>2sigma(I)]**	0.0262(6022)
***w*R$_2$ [I>2sigma(I)**	0.0466(6582)

Figure S15. X-Ray structure of complex **5A-H$_2$O** obtained by crystallization from CD_2Cl_2. Ellipsoids are set at 50% probability.

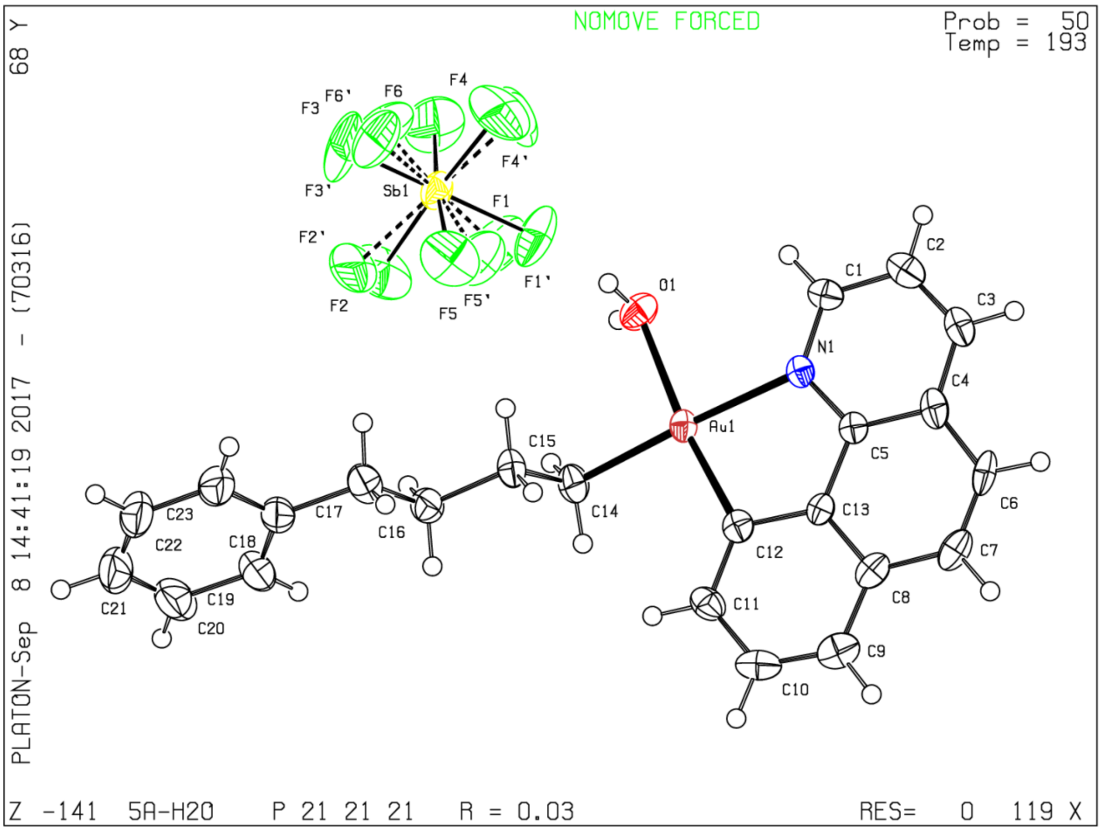

Table S4. Key structural parameters of complex **5A-Cl**.

**Chemical formula**	$C_{23}H_{21}Au_1Cl_1N_1C_1H_1Cl_3$
**fw (g mol^{-1})**	663.20
**Bond precision**	C-C = 0.0055Å
**T (K)**	193
***a* (Å)**	7.4651(4)
***b* (Å)**	9.8244(5)
***c* (Å)**	16.9630(9)
**α (deg.)**	98.935(2)
**β (deg.)**	91.454(2)
**γ (deg.)**	106.868(2)
***V* (Å^3)**	1172.83(11)
**$\rho_{calcd.}$ (g cm^{-3})**	1.878
**λ (Å)**	0.71073
**Mu (mm-1)**	6.739
**F000**	640.0
**F000'**	637.48
**h, k, lmax**	10,13,23
**Nref**	6872
**R_1 [I>2sigma(I)]**	0.0247(6265)
**wR_2 [I>2sigma(I)]**	0.0583(6865)

Figure S16. X-Ray structure of complex **5A-Cl** obtained by crystallization from $CHCl_3$. Ellipsoids are set at 50% probability.

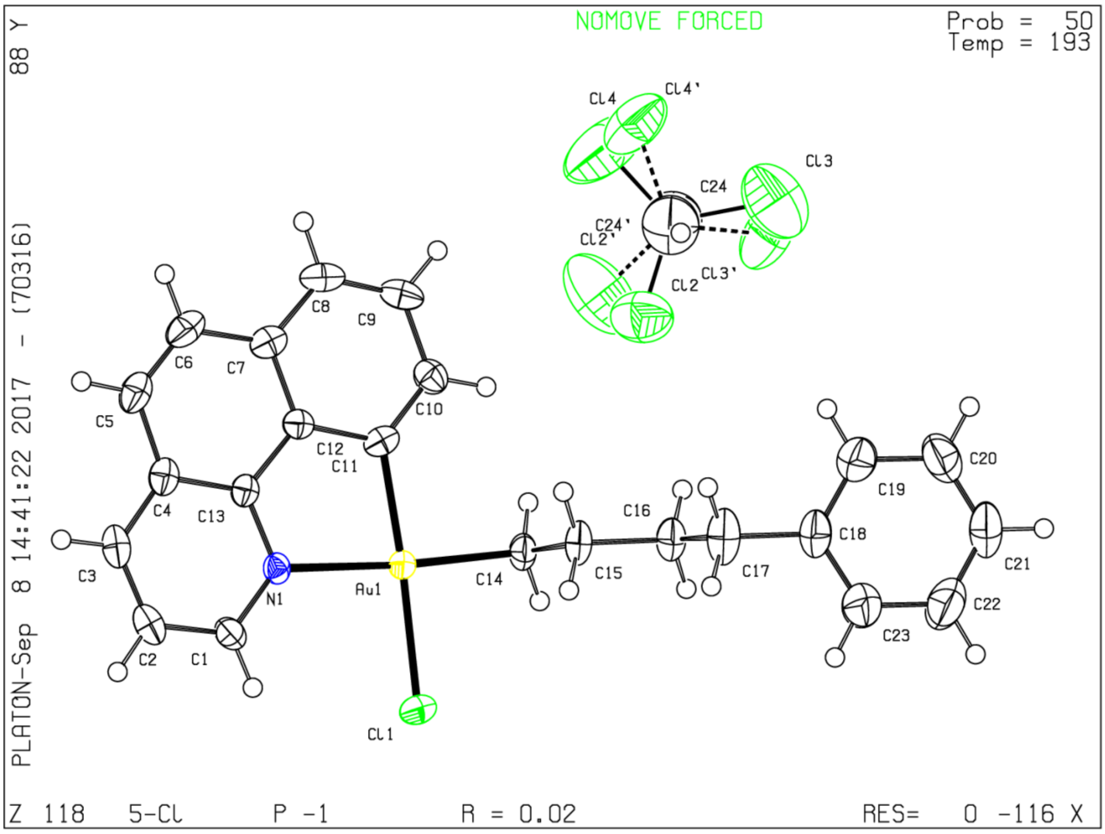

Supplement: Supplementary file 2 [file SC-009-C7SC04899H-s002.pdf]
